# GOunder/Desmoid Tumor Research Foundation DEsmoid Symptom/Impact Scale (GODDESS^©^): psychometric properties and clinically meaningful thresholds as assessed in the Phase 3 DeFi randomized controlled clinical trial

**DOI:** 10.1007/s11136-023-03445-7

**Published:** 2023-06-22

**Authors:** Mrinal M. Gounder, Thomas M. Atkinson, Timothy Bell, Christina Daskalopoulou, Pip Griffiths, Moriah Martindale, L. Mary Smith, Allison Lim

**Affiliations:** 1 Memorial Sloan Kettering Cancer Center, New York, NY USA; 2 SpringWorks Therapeutics, Inc., Stamford, CT USA; 3 IQVIA, Patient-Centered Solutions, Athens, Greece; 4 Patient-Centered Solutions, IQVIA, Paris, France

**Keywords:** Patient-reported outcomes, Desmoid tumor, Aggressive fibromatosis, Psychometric properties, Disease symptom assessment, Rare diseases

## Abstract

**Purpose:**

The GODDESS^©^ tool was developed to assess Desmoid Tumor/Aggressive Fibromatosis (DT/AF) symptom severity and impact on patients’ lives. This study evaluated GODDESS^©^’s cross-sectional and longitudinal measurement properties.

**Methods:**

The Phase 3, randomized placebo-controlled, DeFi study (NCT03785964) of nirogacestat in DT/AF was used to assess GODDESS^©^’s reliability, construct validity, responsiveness, and estimate of meaningful change thresholds (MCTs). Other patient-reported outcome (PRO) measures included Patient Global Impression of Severity (PGIS) in DT/AF symptoms, EORTC QLQ-C30, Brief Pain Inventory Short Form, and PROMIS Physical Function short-form 10a v2.0 plus 3 items.

**Results:**

DeFi participants (*N* = 142) had a median age of 34 years (range: 18–76) and were mostly female (64.8%), with extra-abdominal (76.8%) or intra-abdominal tumors (23.2%). The GODDESS^©^ symptom/impact scales showed internal consistency at baseline, cycles 4 and 7 (Cronbach’s *α* > 0.70) and test–retest reliability (intra-class correlation coefficient > 0.85). GODDESS^©^ scales correlated moderately to highly with PRO measures capturing similar content and differentiated among PGIS and Eastern Cooperative Oncology Group groups. GODDESS^©^ scales detected improvement over time. For the total symptom score, a 1.30-point decrease was estimated as the within-person MCT and a 1.00-point decrease as the between-group MCT. For the physical functioning impact score, estimated within- and between-group MCTs were 0.60-point and 0.50-point decreases, respectively. Few participants exhibited symptom worsening.

**Conclusion:**

GODDESS^©^ was found to be reliable, valid, responsive, and interpretable as a clinical trial endpoint in the pooled sample of DT/AF patients. Estimated MCTs can be used to define responders and assess group-level differences in future, unblinded, efficacy analyses.

**Trial registration number and registration date:**

NCT03785964; December 24, 2018.

**Supplementary Information:**

The online version contains supplementary material available at 10.1007/s11136-023-03445-7.

## Plain English summary

Desmoid tumors are associated with significant symptoms that affect patients’ quality of life. The effect of treatment on patient-reported outcomes is as important as its effect on the tumor. The efficacy and safety of nirogacestat have been evaluated in an appropriately designed clinical study. Although patient-reported outcome(s) may be included in a clinical study, these outcomes are infrequently included in drug labeling for oncology drugs primarily due to challenges related to the reliability and validity of the measurements. The GODDESS tool was developed to assess Desmoid Tumor/Aggressive Fibromatosis (DT/AF) symptom severity and their impact on patients’ lives. This paper presents the results of the analysis of patient-reported outcomes in the study of nirogacestat. GODDESS was found to be dependable, valid, responsive, and interpretable as a means of examining the effect of treatment on patient-reported outcomes.

## Introduction

Desmoid tumor (DT), also known as aggressive or deep fibromatosis [1], is characterized by the development of non-metastasizing, locally aggressive connective-tissue neoplasms and may cause significant morbidity [2]. DT is diagnosed in approximately 1000 to 1650 people in the United States annually, constituting less than 3% of all soft-tissue tumors [3–5]. DT can arise in almost any soft tissue but most typically are found in the abdominal wall, intra-abdominal cavity, and extremities [6].

Although DT mortality is low, DT is associated with significant symptoms and impact on patients’ health-related quality of life (HRQoL), particularly when the tumor(s) grow larger regardless of the location [3, 7]. The most frequently reported symptoms associated with DT include pain, soreness, or tingling (caused by the tumor pressing on nearby nerves, muscles, or blood vessels), difficulty moving the arms or hands, limping, or other difficulties moving the legs or feet [4, 8]. Intra-abdominal DT may also cause bloating, constipation, abdominal pain, and/or intestinal obstruction. Improvement of symptomatology and limiting the effects on daily living are highly important to patients with DT [8]; however, no Food and Drug Administration (FDA)-approved therapies are available for DT.

Treatment of DT requires multidisciplinary team collaboration to create an overall treatment plan with the goal of improving both clinical markers, such as progression-free survival and objective response, as well as patient-relevant outcomes such as desmoid-specific symptoms (e.g., pain), functioning, and overall HRQoL [9–11]. Possible treatment options include systemic therapies, locoregional therapies (e.g., surgery), and/or active surveillance [12]. The presence of a tumor, in addition to the active treatment used to control the tumor, may cause side effects, as well as emotional, social, and financial effects. Hence, patient-reported outcome (PRO) measures are needed to appropriately assess the patient experience. Despite the importance of the patient experience in the overall treatment plan, PRO measures rarely progress into the FDA drug labeling for oncology drugs primarily due to the methodological challenges questioning the accurate capture of patient experience, for instance, lack of pre-specification or multiplicity adjustment, or existence of appropriate PRO instruments for a specific disease state [13, 14]. The absence of PRO measure data in labeling limits a holistic understanding of the treatment benefit-risk and may compromise fully informed treatment decisions. Following the 21st Century Cures Act of 2016, the FDA issued a guidance aiming to advance the collection of patient experience data for regulatory decision-making [15].

The Gounder/DTRF Desmoid Symptom/Impact Scale (GODDESS^©^) was developed to assess patient-reported signs and symptoms associated with DT and their impact on functioning and daily living, with the intent to better capture the patient experience and treatment effects in DT and eventual use as an explanatory endpoint in clinical trials. To date, development of the GODDESS^©^ included qualitative research [8], but evidence of the instrument’s psychometric properties, including the interpretation of clinically meaningful within-person and between-group changes, was lacking. This evidence is needed to enable researchers to make informed decisions on the appropriateness of GODDESS^©^ as a clinical trial efficacy endpoint.

The objectives of this study were to assess GODDESS^©^ cross-sectional and longitudinal measurement properties, including assessment of clinically meaningful, within-person and between-group change in score, using data from a clinical study within the instrument’s context of use.

## Materials and methods

### Data source

Analyses were conducted using blinded data from the Phase 3, double-blind, randomized, placebo-controlled study of nirogacestat in adults with desmoid tumor/aggressive fibromatosis (DT/AF) (DeFi; NCT03785964). Patients were administered nirogacestat or placebo tablets twice daily continuously in 28-day cycles. Eligible patients were ≥ 18 years old, with histologically confirmed DT/AF that progressed by ≥ 20% per Response Evaluation Criteria In Solid Tumors (RECIST) v1.1 within 12 months of screening, and an Eastern Cooperative Oncology Group (ECOG) Performance Status (PS) score ≤ 2. A 1:1 randomization allocation was stratified based on tumor location: intra-abdominal (including mesentery/pelvis) and extra-abdominal (including head/neck, para-spinal, extremities, abdominal wall, chest wall, and other). DeFi was conducted in accordance with Good Clinical Practice; all study materials were approved by the appropriate ethics body at each participating center. Informed consent was obtained prior to any study procedures and follows ICH GCP/CFR guidelines. Consent included the collection and use of NIR-DT-301 study data, which were used for the validation of the GODDESS^©^ tool. See further details in Appendix 3 of the supplementary material [16].

### Patient-reported outcome assessments

The GODDESS^©^ tool was developed by Memorial Sloan Kettering Cancer Center (MSK) and Desmoid Tumor Research Foundation (DTRF) to measure signs and symptoms of DT (Desmoid Tumor Symptom Scale: DTSS) and their impact on patients’ lives (Desmoid Tumor Impact Scale: DTIS) based on previously conducted content validity related work [8].

DTSS comprises 11 items assessing key signs and symptoms severity including pain, fatigue, swelling, muscle weakness, difficulty moving (items 1–7); a question referring to tumor location (item 8); and intra-abdominal-specific signs/symptoms (items 9–11) administered only to those reporting intra-abdominal tumor location in item 8. DTIS comprises 17 items assessing symptoms impact on functioning and daily living. DTSS items 1–7 and 9–11 are evaluated on an 11-point numeric rating scale (NRS) from 0 to 10 to measure severity from “none” to “as bad as you can imagine,” with a 24-h recall period; item 8 asks for the specific location of the desmoid tumor. DTIS items are evaluated either on a 5-point Likert scale ranging from “none of the time” to “all of the time” to measure frequency (items 1–9), or an 11-point NRS from 0 to 10 to measure severity from “none” to “as bad as you can imagine” (items 10–17), with a 7-day recall period. Higher scores indicate more severe symptomatology/impact. GODDESS^©^ is available in 20 languages.

Other PRO measures used in the analysis included the Brief Pain Inventory Short Form (BPI-SF) [17], which assesses clinical pain severity and pain interference with feelings and functions; the Patient-Reported Outcomes Measurement Information System Physical Function (PROMIS-PF) short-form 10a version 2.0 plus 3 additional items [18], which assesses various self-reported capability of physical activities; the European Organisation for Research and Treatment of Cancer Quality of Life Questionnaire-Core 30 (EORTC QLQ-C30) version 3.0 [19], which assesses cancer patients’ HRQoL; the Patient Global Impression of Severity (PGIS) and the Patient Global Impression of Change (PGIC) referring specifically to desmoid symptoms severity and overall status [20], respectively.

All PRO measures were completed by the patients using home electronic PRO (ePRO) devices at screening, baseline (i.e., Cycle 1), and in 28-day cycles thereafter. For full details of the PRO measure assessment schedule, see Appendix 4 of the supplementary material.

### Analyses

Measurement properties of the PRO tools were evaluated by using measurements at baseline, cycle 4, and cycle 7 from the DeFi study. The blinded data were pooled across treatment arms for these validation analyses. Baseline demographic and clinical variables were described using descriptive statistics (e.g., mean, standard deviation (SD), median) for quantitative variables, and percentage and frequency for categorical variables.

Completion rate from baseline was calculated as the number of patients expected at each timepoint (excluding subject discontinuations) divided by the number of patients still on trial at the analysis timepoint. A summary DTSS score over a week was defined as the average of the daily diary score over the 7-day period if the patient completed ≥ 4 of 7 days, otherwise the score was considered missing.

Inter-item correlations at baseline were assessed using Spearman and polychoric correlation coefficients for the 11-point NRS item pairs and the 5-point Likert scale items, respectively. Values > 0.4 provided support for combining items into a multi-item scale, whereas pairs of items with coefficients > 0.9 and/or < 0.3 were considered for further scrutiny when developing the scoring algorithm [21–23]. Confirmatory factor analyses (CFA) were performed to further evaluate the structure (Appendix 1).

Internal consistency reliability, reflecting the degree to which a set of items in the same scale co-vary, was assessed at baseline, cycles 4 and 7 using Cronbach’s alpha coefficients with 95% confidence intervals (CIs); values ≥ 0.70 are considered to represent acceptable reliability [24]. Test–retest reliability, i.e., the repeatability of scores over a time period in patients who are not expected to experience change, was assessed among stable subjects, defined as subjects with no change in PGIS responses between baseline and cycle 2, using 2-way mixed, absolute agreement, single-measure, intra-class correlation coefficients (ICCs) between the two assessments [25]. Values of 0.50–0.90 are considered to represent moderate to good reliability and values > 0.90 excellent reliability [26].

Construct validity was assessed by examining convergent and known-groups validity. Convergent validity, referring to how well constructs that theoretically should be related to each other are observed to be related, was evaluated at baseline by correlation coefficients between DTSS and DTIS and concepts captured from other PRO instruments (i.e., DTSS and BPI-SF Pain Severity items and overall Pain Severity scale, PROMIS-PF short-form 10a score, EORTC QLQ-C30 Global Health Status, symptom scales, and appetite loss; DTIS and BPI-SF Interference scale, 3 PROMIS items, EORTC QLQ-C30 functional scales and insomnia). Correlation coefficients ≥ 0.40 are considered evidence of convergent validity [22]. Known-groups validity, demonstrating the degree to which DTSS and DTIS scores can distinguish among groups of subjects hypothesized to be different in the concept of interest, was evaluated by investigating whether the distributions of DTSS and DTIS varied by groups defined by PGIS (response categories) and ECOG PS (0 versus 1).

### Responsiveness and interpretation of meaningful within-person change in score

Responsiveness reflects the ability of an instrument to detect changes in groups of patients who have changed in the measured concept. Change from baseline to cycles 4 and 7 for DTSS and DTIS was evaluated within groups of patients who had changed according to PGIS, using analysis of covariance (ANCOVA) and magnitude of change via effect sizes (ES) (within-group: mean change from baseline/SD_baseline_; between-group: mean change from baseline between two groups/SD_pooled_). Cohen’s ES rules were used for interpretation (i.e., 0.20 = small, 0.50 = moderate, and 0.80 = large) [27].

Meaningful change was evaluated both at within-person level (i.e., the amount of change a person would report to indicate that a relevant benefit has been experienced) and between-group level (i.e., the difference in scores between treatment and comparator groups considered clinically meaningful). Of note, within-person thresholds can be used to define responders for statistical tests of proportions improved in each treatment group, whereas between-group thresholds support statistical inference of mean change between treatment and comparator groups and are used to assess the magnitude of the observed difference. To define meaningful change thresholds (MCTs) in DTSS and DTIS, anchor-based and distribution-based methods were evaluated and supported by visual displays of change score distribution using empirical cumulative distribution function (eCDF) curves. Firstly, the appropriateness of anchors (i.e., PGIS, PGIC) was tested by correlating them with DTSS and DTIS change scores from baseline to cycles 4 and 7; correlations > 0.30 were considered desirable [28]. Anchor-based methods included descriptive and modeling approaches (mixed models repeated measures [MMRM], Appendix 2) of the change scores from baseline. Distribution-based estimates included the half SD of the baseline score and the standard error of measurement, i.e., SD_baseline_ × sqrt[1−test–retest reliability coefficient]. MCTs at the within-person level were evaluated based on the anchor-based estimates if the latter exceeded the relevant distribution-based estimates and the absolute upper 95% CI of the anchor ‘no change’ group. MCTs at the between-group level were also estimated based on anchor-based estimates and more specifically on the mean change between minimally improved patients and those exhibiting ‘no change.’

### Results

Of 142 total patients randomized in DeFi, 109 (76.8%) had extra-abdominal disease and 33 (23.2%) had intra-abdominal disease. At baseline, median age was 34 years, and the majority were female (64.8%), white race (83.1%), non-Hispanic/Latino ethnicity (85.9%), and enrolled in North America (68.3%) (Table 1).

**Table 1:** Demographic and baseline clinical information

Characteristic	Overall
Type of tumor	
Total, *N*	142
Intra-abdominal, *n* (%)	33 (23.2%)
Extra-abdominal, *n* (%)	109 (76.8%)
Gender	
Total, *N*	142
Male, *n* (%)	50 (35.2%)
Female, *n* (%)	92 (64.8%)
Age at time of consent (year)	
*N*	142
Mean (SD)	37.2 (13.6)
Median	34.0
Min/max	18.0/76.0
Q1/Q3	27.0/46.0
Skewness	0.8
Race	
Total, *N*	142
White, *n* (%)	118 (83.1%)
Black or African American, *n* (%)	9 (6.3%)
Asian, *n* (%)	4 (2.8%)
Other, *n* (%)	11 (7.7%)
Ethnicity	
Total, *N*	142
Hispanic or Latino, *n* (%)	10 (7.0%)
Not Hispanic or Latino, *n* (%)	122 (85.9%)
Unknown, *n* (%)	3 (2.1%)
Not reported, *n* (%)	7 (4.9%)
Geographic region	
Total, *N*	142
Europe, *n* (%)	45 (31.7%)
North America, *n* (%)	97 (68.3%)

*N* number of cases, *y* years, *SD* standard deviation, *min* minimum, *max* maximum

### Completion and distribution

One patient was excluded from analysis due to incongruous baseline dates. For the DTSS, a high completion rate was observed at baseline weekly period (i.e., 68.8% from day 1 to 93.6% to day 7). Completion rates dropped in later cycles, although with most days remaining above 65%. For the DTIS, a high completion rate was observed at baseline (92.9%) and at later cycles (65.4% at cycle 4, 67.0% at cycle 7).

**Fig. 1:**
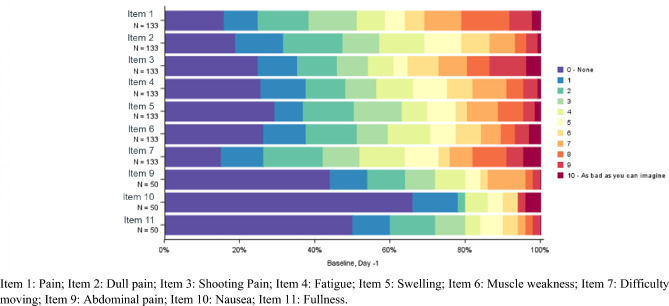
DTSS item distribution at baseline: day -1

Patients showed low symptomatology at baseline with mostly ceiling effects (≥ 20% patients reported the “best” possible score 0 = None, i.e., could not improve) and a good distribution of remaining response options in all DTSS items. Items referring specifically to intra-abdominal symptoms were only answered by a subset, i.e., those with intra-abdominal tumors. These items had the highest percentage of ceiling effects (e.g., ~ 70% of patients reported “None” for item 10: “Nausea”) (Fig. 1). In general, symptomatology improvement was observed in the post-baseline cycles, as most patients reported no symptoms (Supplementary Figs. S2, S3).

**Fig. 2:**
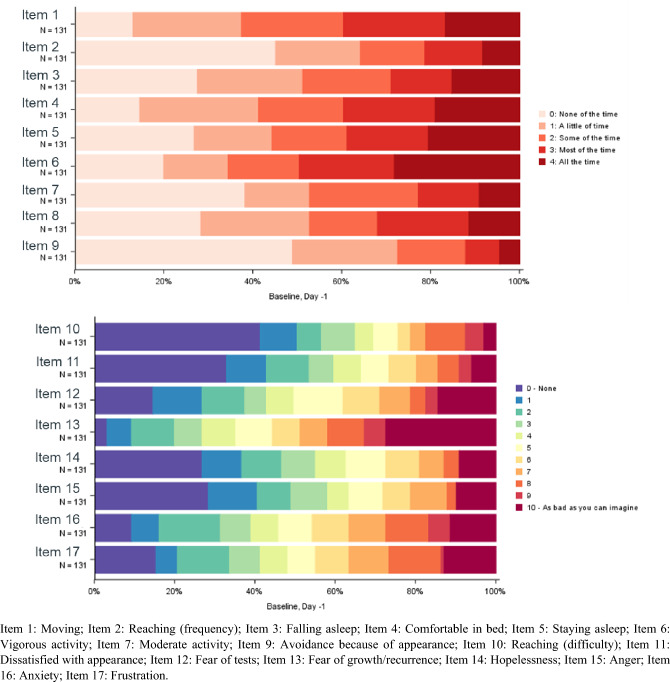
DTIS Item distribution at baseline: day-1

Patients reported low impact of disease on functioning and daily living at baseline, as most DTIS items exhibited floor effects (> 20% reported no impact of the symptom). An exception to this was observed for the mobility items (i.e., item 1: difficulty moving, and item 4: comfortable in bed), as less than 20% reported no impact. Items reflecting mental concerns (e.g., item 12: fear tests, item 13: fear of recurrence/growth, item 16: anxiety, and item 17: frustration) reported high percentages (≥ 20%) of the worst impact option (Fig. 2). Most DTIS items showed improvement in the post-baseline cycles (Supplementary Fig. S4–S7).

### Structural validity

DTSS inter-item correlations were generally moderate to large (0.50 ≤ *r* < 0.90), indicating that a total score is plausible. Larger correlations were mostly observed among items 1–7, supporting the combination of these into a total symptom scale. The largest correlations were observed among items 1–3, providing evidence of a domain reflecting pain. In addition, moderate-to-strong correlations were observed among items 5–7, supporting an extra-abdominal domain. In contrast, items 9–11, reflecting intra-abdominal symptoms (and answered by a subsample of the initial population [*n* = 50]), showed lower correlations (Table 2).

**Table 2:** Item-to-item correlation: GODDESS^©^ DTSS items at baseline

Item	Item 2: dull pain	Item 3: shooting pain	Item 4: fatigue	Item 5: swelling	Item 6: muscle weakness	Item 7: difficulty moving	Item 9: abdominal pain	Item 10: nausea	Item 11: fullness
Item 1: pain	(132) 0.88	(132) 0.90	(132) 0.69	(132) 0.69	(132) 0.73	(132) 0.77	(50) 0.76	(50) 0.50	(50) 0.53
Item 2: dull pain		(132) 0.83	(132) 0.69	(132) 0.69	(132) 0.69	(132) 0.73	(50) 0.73	(50) 0.53	(50) 0.60
Item 3: shooting pain			(132) 0.65	(132) 0.69	(132) 0.70	(132) 0.74	(50) 0.74	(50) 0.37	(50) 0.37
Item 4: fatigue				(132) 0.62	(132) 0.63	(132) 0.70	(50) 0.49	(50) 0.69	(50) 0.73
Item 5: swelling					(132) 0.76	(132) 0.71	(50) 0.48	(50) 0.47	(50) 0.43
Item 6: muscle weakness						(132) 0.89	(50) 0.57	(50) 0.57	(50) 0.47
Item 7: difficulty moving							(50) 0.47	(50) 0.53	(50) 0.48
Item 9: abdominal pain								(50) 0.48	(50) 0.56
Item 10: nausea									(50) 0.77

^©^ 2022 Memorial Sloan Kettering Cancer Center, et al. All rights reserved. The content and design of this questionnaire are protected by US and international copyright laws. This questionnaire or any portion thereof may not be reproduced, distributed, altered, or used in any manner without. prior written consent from Memorial Sloan Kettering Cancer Center. If you require further information on a permitted use or a license to use any content, email MarComReview@mskcc.org

*DTSS* desmoid tumor symptom scale

Moderate inter-item correlations were observed among DTIS items 1, 2, 6, 7, 8 and 10, supporting combination of these into a domain reflecting physical functioning impact. However, item 10 was strongly correlated with item 2 (i.e., *r* > 0.90), suggesting potentially redundant content (both items examined the ‘reaching up’ impact). Moderate inter-item correlations were also observed among items 3–5, supporting the creation of a domain reflecting sleep impact. Strong correlations (*r* > 0.70) were observed among items 12–17, providing support to create a domain of emotional impact. Items 9 and 11 (‘appearance’ impact) were not highly correlated with other items, indicating they may not belong to a specific domain (Table 3).

**Table 3:** Item-to-item correlation: GODDESS^©^ DTIS items at baseline

Item	Item 2: reaching (frequency)	Item 3: falling asleep	Item 4: comfortable in bed	Item 5: staying asleep	Item 6: vigorous activity	Item 7: moderate activity	Item 8: accomplished less	Item 9: avoidance because of appearance	Item 10: reaching (difficulty)
Item 1: moving	(131) 0.66	(131) 0.56	(131) 0.71	(131) 0.45	(131) 0.77	(131) 0.78	(131) 0.66	(131) 0.49	(131) 0.57
Item 2: reaching (frequency)		(131) 0.74	(131) 0.72	(131) 0.56	(131) 0.67	(131) 0.73	(131) 0.78	(131) 0.62	(131) 0.93
Item 3: falling asleep			(131) 0.88	(131) 0.84	(131) 0.56	(131) 0.65	(131) 0.56	(131) 0.53	(131) 0.59
Item 4: comfortable in bed				(131) 0.80	(131) 0.66	(131) 0.72	(131) 0.58	(131) 0.52	(131) 0.59
Item 5: staying asleep					(131) 0.47	(131) 0.53	(131) 0.49	(131) 0.44	(131) 0.47
Item 6: vigorous activity						(131) 0.90	(131) 0.83	(131) 0.59	(131) 0.56
Item 7: moderate activity							(131) 0.88	(131) 0.58	(131) 0.59
Item 8: accomplished less								(131) 0.66	(131) 0.63
Item 9: avoidance because of appearance									(131) 0.51
Item 10: reaching (difficulty)									
Item 11: dissatisfied with appearance									
Item 12: fear of tests									
Item 13: fear of recurrence/growth									
Item 14: hopelessness									
Item 15: anger									
Item 16: anxiety									

Item	Item 11: dissatisfied with appearance	Item 12: fear of tests	Item 13: fear of recurrence/growth	Item 14: hopelessness	Item 15: anger	Item 16: anxiety	Item 17: frustration
Item 1: moving	(131) 0.40	(131) 0.32	(131) 0.32	(131) 0.41	(131) 0.29	(131) 0.30	(131) 0.45
Item 2: reaching (frequency)	(131) 0.48	(131) 0.39	(131) 0.33	(131) 0.38	(131) 0.34	(131) 0.42	(131) 0.50
Item 3: falling asleep	(131) 0.41	(131) 0.32	(131) 0.39	(131) 0.45	(131) 0.46	(131) 0.43	(131) 0.54
Item 4: comfortable in bed	(131) 0.48	(131) 0.37	(131) 0.47	(131) 0.47	(131) 0.40	(131) 0.40	(131) 0.53
Item 5: staying asleep	(131) 0.41	(131) 0.28	(131) 0.37	(131) 0.34	(131) 0.31	(131) 0.38	(131) 0.47
Item 6: vigorous activity	(131) 0.49	(131) 0.34	(131) 0.41	(131) 0.43	(131) 0.29	(131) 0.33	(131) 0.48
Item 7: moderate activity	(131) 0.45	(131) 0.29	(131) 0.38	(131) 0.36	(131) 0.25	(131) 0.32	(131) 0.44
Item 8: accomplished less	(131) 0.54	(131) 0.35	(131) 0.40	(131) 0.43	(131) 0.35	(131) 0.39	(131) 0.48
Item 9: avoidance because of appearance	(131) 0.81	(131) 0.39	(131) 0.37	(131) 0.37	(131) 0.41	(131) 0.38	(131) 0.45
Item 10: reaching (difficulty)	(131) 0.48	(131) 0.37	(131) 0.28	(131) 0.37	(131) 0.30	(131) 0.36	(131) 0.44
Item 11: dissatisfied with appearance		(131) 0.45	(131) 0.46	(131) 0.46	(131) 0.41	(131) 0.42	(131) 0.48
Item 12: fear of tests			(131) 0.71	(131) 0.64	(131) 0.52	(131) 0.70	(131) 0.56
Item 13: fear of recurrence/growth				(131) 0.65	(131) 0.60	(131) 0.68	(131) 0.61
Item 14: hopelessness					(131) 0.76	(131) 0.69	(131) 0.76
Item 15: anger						(131) 0.65	(131) 0.77
Item 16: anxiety							(131) 0.71

^©^ 2022 Memorial Sloan Kettering Cancer Center, et al. All rights reserved. The content and design of this questionnaire are protected by US and international copyright laws. This questionnaire or any portion thereof may not be reproduced, distributed, altered, or used in any manner without. prior written consent from Memorial Sloan Kettering Cancer Center. If you require further information on a permitted use or a license to use any content, email MarComReview@mskcc.org

*DTIS* desmoid tumor impact scale

Our hypotheses were further confirmed via CFA models (Appendix 1). Considering the above, a DTSS total symptom score based on items 1–7 (i.e., excluding the specific items for patients with intra-abdominal tumors) and individual domain scores reflecting pain (items 1–3), extra-abdominal symptoms (items 5–7), and intra-abdominal symptoms (items 9–11) were created. Total symptom and individual domain scores were created by averaging the daily scores to constitute a daily score, and weekly scores were created by averaging the daily score of at least 4 out of 7 days before each timepoint. For the DTSS total score, the pain domain score (average of items 1–3) was averaged with items 4–7. For DTIS, three individual domain scores were created reflecting impact on physical functioning (items 1, 2, 6, 7, 8), sleep (items 3–5), and emotional (items 12–17). Items 9, 10, and 11 were considered standalone scores and did not undergo further psychometric assessment. DTSS total symptom and individual domains, and DTIS emotional impact domain scores, range from 0 to 10. DTIS physical functioning and sleep domain scores range from 0 to 4 (Supplementary Table S1).

**Fig. 3:**
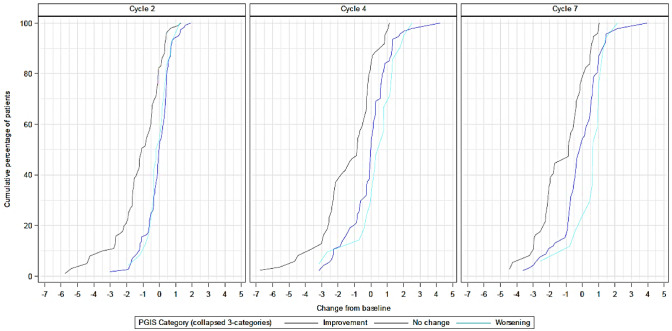
Empirical cumulative distribution function of DTSS by PGIS over time

Internal consistency reliability of the DTSS total symptom score was above the conventional threshold of 0.70 at both baseline (0.95; 95% CI 0.94; 0.96) and cycle 7 (0.96, 95% CI 0.95; 0.98). Similar findings were observed for the DTSS individual domains. Internal consistency reliability was also above the threshold for all DTIS domains (Supplementary Table S2). Test–retest reliability, calculated in subjects with ‘no change’ in PGIS scores between baseline and cycle 2, was > 0.90 for the DTSS total symptom and domain scores and > 0.85 for all DTIS domains (Fig. 3).

Construct validity was well demonstrated by moderate-to-strong correlations of DTSS total symptom and individual domain scores (i.e., pain, extra-abdominal, intra-abdominal) with similar concepts from other PRO measures as hypothesized. For example, stronger correlations were found with measures of more highly related constructs (such as the “BPI pain at its worst in the last 24 h”; Total *r* = 0.74; pain domain *r* = 0.87; extra-abdominal domain *r* = 0.64; intra-abdominal domain *r* = 0.58) than more distally related constructs (such as the “EORTC QLQ-C30 Appetite Loss Scale”; Total *r* = 0.18; pain domain *r* = 0.19; extra-abdominal domain *r* = 0.13; intra-abdominal domain *r* = 0.34). Further relationships are shown in Supplementary Table S3. Similarly, construct validity was also supported for the DTIS domains using similar hypotheses. For example, stronger correlations were found between the “PROMIS-PF short form Bend or Twist Your Back” item and the physical functioning domain (*r* = − 0.69) than with the sleep domains (− 0.39) or the emotional domain (− 0.37; further relationships displayed in Supplementary Table S4). Construct validity was further demonstrated by comparing DTSS (total symptom and individual domains) and DTIS (individual domains) scores among groups hypothesized to be different at baseline. Higher mean scores, indicating more severe symptomatology and impact, were observed in groups defined using PGIS (higher responses indicating more severe severity), and ECOG PS (with higher score indicating a lower level of functionality) (Supplementary Fig. S1).

### Responsiveness and interpretation of meaningful within-person change in score

The mean score change difference among groups of patients specified as PGIS improved, stable, or worsened followed the expected pattern in DTSS and DTIS. Mean DTSS total change score for these PGIS groups were: − 1.52 for improved, − 0.23 for stable, and 0.76 for worsened. Results of the same direction were identified for the DTSS pain, extra-abdominal, and intra-abdominal domains, and the DTIS physical functioning, sleep, and emotional impact domains. ES for the between-groups were mostly moderate to large for the improved group, supporting ability to detect change (Table 4). ES were mostly small-to-moderate for the worsened group. The latter could also be due to the limited number of patients deteriorated throughout the study. For this reason, MCTs are estimated only for improvement.

**Table 4:** Ability to Detect Change: Change in DTSS and DTIS scores by PGIS groups from Baseline to Cycle 4

Scale	Score	PGIS group	N	LS mean (SE)	95%CI of LS mean	Within-groups effect size	Between-groups effect size	*P*-value
DTSS	Total symptom	Improvement	30	− 1.52 (0.23)	[− 1.98; − 1.05]	1.03	1.20	
		No change	33	− 0.23 (0.22)	[− 0.67; 0.21]	0.10		
		Worsening	10	0.76 (0.40)	[− 0.03; 1.56]	0.44	0.71	< 0.001
	Pain	Improvement	30	− 2.36 (0.31)	[− 2.97; − 1.75]	1.10	1.08	
		No change	33	− 0.63 (0.29)	[− 1.21; − 0.05]	0.33		
		Worsening	10	1.36 (0.53)	[0.31; 2.41]	1.93	1.48	< 0.001
	Extra-abdominal	Improvement	30	− 1.58 (0.28)	[− 2.14; − 1.02]	0.96	1.07	
		No change	33	− 0.29 (0.27)	[− 0.82; 0.25]	0.14		
		Worsening	10	0.73 (0.48)	[− 0.22; 1.68]	0.29	0.57	< 0.001
	Intra-abdominal	Improvement	9	− 0.54 (0.39)	[− 1.34; 0.26]	0.33	0.84	
		No change	14	0.42 (0.32)	[− 0.24; 1.07]	0.55		
		Worsening	5	0.36 (0.56)	[− 0.79; 1.50]	0.04	0.47	0.159
DTIS	Physical functioning	Improvement	33	− 0.68 (0.10)	[− 0.88; − 0.47]	1.12	1.04	
		No change	37	− 0.16 (0.10)	[− 0.35; 0.03]	0.24		
		Worsening	10	0.40 (0.18)	[0.03; 0.77]	0.59	1.00	< 0.001
	Sleep	Improvement	33	− 0.51 (0.13)	[− 0.77; − 0.26]	0.60	0.64	
		No change	37	− 0.07 (0.12)	[− 0.31; 0.17]	0.07		
		Worsening	10	0.36 (0.23)	[− 0.10; 0.83]	0.49	0.65	0.003
	Emotional	Improvement	33	− 1.74 (0.29)	[− 2.32; − 1.17]	1.01	0.93	
		No change	37	− 0.40 (0.27)	[− 0.94; 0.15]	0.15		
		Worsening	10	− 0.02 (0.52)	[ − 1.06; 1.01]	0.03	0.12	0.001

For a DTSS scale summary score calculation, it was assumed that at least 4 days out of 7 were available. If sufficient data were not available at baseline, data from the screening period (if available) were used instead. The between-groups effect size was calculated as the mean group difference divided by the pooled standard deviation at baseline. The within- groups effect size of change over time was calculated as the mean change in score over time divided by the baseline standard deviation for that group.

Effect sizes were judged in terms of Cohen's effect size rules: *N* number of cases, *LS mean* least squares mean, *SE* standard error, *CI* confidence interval, *DTSS* desmoid tumor symptom scale, *DTIS* desmoid tumor impact scale, *PGIS* patient global impression of severity

Correlation coefficients between DTSS total and individual domain, and DTIS individual domains change scores, with PGIS, were above the recommended threshold of 0.30 for all scale scores except for DTSS intra-abdominal domain. For the latter, PGIC was selected as the more appropriate anchor (Supplementary Table S5); however, the recommended threshold was met only at cycle 4 (Table 5).

**Table 5:** **T**hreshold estimates for within-person improvement in DTSS and DTIS scores

Scale	Score	MMRM estimates^a^	PGIS/PGIC^a^ Cycle 4 Median [mean (SD)]	PGIS/PGIC^a^ Cycle 7 Median [mean (SD)]	0.5 SD of baseline scores	SEM	Recommended within-person threshold
DTSS	Total symptom	1.33	1.51 [1.64 (1.59)]	1.27 [1.32 (1.35)]	1.23	0.36	1.30
	Pain	2.07	2.24 [2.58 (2.35)]	2.00 [2.25 (1.99)]	1.34	0.67	2.00
	Extra-abdominal	1.41	1.25 [1.71 (1.79)]	1.56 [1.56 (1.60)]	1.31	0.41	1.40
	Intra-abdominal	0.65	0.10 [0.00 (1.14)]	0.03 [0.39 (2.47)]	0.95	0.50	1.00
DTIS	Physical functioning	0.56	0.60 [0.73 (0.65)]	0.60 [0.77 (0.73)]	0.59	0.36	0.60
	Sleep	0.44	0.33 [0.56 (0.93)]	0.33 [0.84 (1.27)]	0.65	0.50	0.70
	Emotional impact	1.75	2.17 [1.90 (1.89)]	3.00 [2.73 (2.22)]	1.41	1.12	1.80

*MMRM* mixed model repeated measures, *SD* standard deviation, *SEM* standard error of measurement, *PGIS* patient global impression of severity, *PGIC* patient global impression of change

^**a**^For Intra-abdominal domain, the PGIC anchor was used

For patients with improvement in the PGIS anchor, change in DTSS total score ranged from 1.27 to 1.64 (mean and median values), including the anchor-model approach estimate (1.33) (Appendix 2). Additionally, distribution-based estimates ranged from 0.41 to 1.20, indicating that change score magnitude is likely larger than the measurement error. Hence, a 1.30-decrease was recommended as the improvement within-person MCT for DTSS total score. Following similar rationale, the recommended improvement within-person MCT for DTSS pain domain score was a 2.00-decrease; for DTSS extra-abdominal domain, a 1.40-decrease, for DTSS intra-abdominal domain, a 1.00-decrease; for DTIS physical functioning, a 0.60-decrease; for DTIS sleep, a 0.70-decrease; and for DTIS emotional, a 1.80-decrease. All thresholds exceeded the upper bound of the 95% CI of the ‘no change’ group (Supplementary Table S6). For the between-group improvement MCTs, change in mean scores between minimally improved and stable patients was calculated as a 0.80-decrease for DTSS total score, a 1.16-decrease for DTSS pain domain score, and a 1.00-decrease for DTSS extra-abdominal domain score. Considering that these are group-level estimations and to avoid underestimation bias, a more conservative between-group improvement estimate of a 1.00-point decrease was recommended for the DTSS total and extra-abdominal domain scores, and a 1.20-point decrease for the DTSS pain domain score. For the DTSS intra-abdominal domain, the number of persons indicating minimal improvement was too low (*n* = 3) to allow confident recommendations. Following similar rationale, the recommended between-group improvement MCT was a 0.50-decrease for DTIS physical functioning and sleep scores, and a 2.00-decrease for DTIS emotional impact score (Supplementary Table S7).

The eCDF curves showed the expected shift to the left and clear differentiation, as seen by consistent separation and generally non-crossing curves, mostly for the improvement group. Curves for ‘no change’ or ‘worsening’ were well separated for DTSS total and individual domain scores, and mostly overlapped for the DTIS individual domain scores. For the DTSS intra-abdominal domain score, no clear differentiation was observed (Supplementary Figs. S8–S13).

## Discussion

This work contributed to the assessment of GODDESS^©^ psychometric properties, including responsiveness and estimates of clinically meaningful within-person and between-group improvement score change in DT patients. Furthermore, scoring algorithms were suggested and all results were evaluated for the identified domains. More specifically, the GODDESS^©^ tool comprises two scales, the DTSS and the DTIS, for which the following scores were suggested: DTSS total score, DTSS pain, DTSS intra-abdominal and extra-abdominal domains; DTIS physical functioning, DTIS sleep, and DTIS emotional domains.

Strengths of this study include the investigation of DTSS and DTIS scales within a blinded, global, interventional study of DT/AF and via ePROs. The latter contribute to more accurate and complete participants’ responses [29]. Descriptive analysis showed ceiling effects revealing a sample of low symptom severity; however, the whole range of item responses was used indicating its appropriateness. Validity and reliability analyses for DTSS and DTIS scales showed that all scores are valid and reliable measures of DT/AF symptomatology and impact. Additionally, recommended thresholds of reliability were met (i.e., ICC > 0.70) for construct validity, including convergent and known-groups validity. Importantly, the responsiveness of the GODDESS^©^ symptom and impact scores suggested a suitable ability to detect change in patients based on those who reported change on the PGIS. Notably, the performance of GODDESS^©^ to assess responsiveness was confirmed regardless of patients’ low symptomatology at baseline. Correlations between change in GODDESS^©^ symptom and impact scores suggested that PGIS was an adequate anchor (*r* > 0.3) for all scores except for DTSS intra-abdominal domain score. The MCT analysis enabled the derivation of within-person and between-group estimates for identifying clinically relevant responders based on improvement. A within-person threshold should be used to define responders; these can then be analyzed via test of proportions to investigate whether more responders exist in the treatment compared to the comparator group. Alternatively, researchers may assess mean change between treatment and comparator groups using statistical comparison. The difference in change scores should be at least as large as the between-group MCT to denote changes important to patients.

Study limitations include the relatively low sample size. Although the sample size was adequate for most analyses and especially for a rare disease, the sample size may be suboptimal for factor analysis. Various rules of thumb for CFA exist, such as typically requiring 10 observations per item [23]. However, going forward, it is recommended to replicate factorial validity in other studies with a sample informed using a simulation-based approach. The work presented here could be used as initial information to inform such a simulation-based sample sizing. Furthermore, the limited sample size, especially within the intra-abdominal domain, in combination with the low sample size experiencing deterioration throughout the study period, did not allow estimation of MCTs for deterioration. However, sample size limitations should be contextualized by considering the rare nature of DT. Although, replication of our findings in other studies with bigger samples is recommended, this may be particularly challenging for this disease area. In addition, although translation studies have already been conducted and GODDESS^©^ is linguistically validated among various cultures and languages, further psychometric validity testing is needed to investigate measurement invariance properties. Finally, it should be noted that like in all scales, the MCT estimation is dependent on the population under study, its baseline characteristics, and the anchors [30]. Therefore, it is recommended that future studies be conducted to also provide MCT estimates in different populations and/or with different anchors.

In conclusion, our study showed that GODDESS^©^ has appropriate psychometric properties. In addition, within-person and between-groups MCTs of improvement for the different symptom and impact domain scales were estimated. These may be used in future efficacy analyses to identify the responders versus non-responders and assess the meaningful efficacy differences between treatment and comparator groups. This study provides a rationale for consideration of the GODDESS^©^ tool to assess DT symptom and impact severity improvement as an endpoint in clinical trials or population research studies.

## Supplementary Information

Below is the link to the electronic supplementary material.

Supplementary file1 (DOCX 1894 kb)
