# Supplementary material for: GOunder/Desmoid Tumor Research Foundation DEsmoid Symptom/Impact Scale (GODDESS©): psychometric properties and clinically meaningful thresholds as assessed in the Phase 3 DeFi randomized controlled clinical trial
Source: Qual Life Res. Author manuscript; Available in PMC 2023 Oct 1. (PMC10474203; doi:10.1007/s11136-023-03445-7)

Supplementary Material associated with “*Gounder M, Atkinson T, Bell T, Daskalopoulou C, Griffiths P, Martindale M, Smith LM and Lim A (2023) GOunder/Desmoid Tumor Research Foundation DEsmoid Symptom/Impact Scale (GODDESS): Psychometric Properties and Clinically Meaningful Thresholds as assessed in the Phase 3 DeFi randomized controlled clinical trial. Quality of Life Research*”

**List of supplementary material (include the supplemental data number and file type)**

1. Supplementary Figure *1* Boxplots of DTSS and DTIS total and domain scores by PGIS at baseline
2. Supplementary Figure 2: DTSS item distribution of daily item responses Cycle 4, Day -1
3. Supplementary Figure *3*: DTSS item distribution of daily item responses Cycle 7, Day -1
4. Supplementary Figure *4* DTIS item distribution of daily item responses (items 1 to 9) Cycle 4, Day -1
5. Supplementary Figure *5*: DTIS item distribution of daily item (items 1 to 9) Cycle 7, Day -1
6. Supplementary Figure *6*: DTIS item distribution of daily item responses (items 10 to 17) Cycle 4, Day -1
7. Supplementary Figure *7*: DTIS item distribution of daily item responses (items 10 to 17) Cycle 7, Day -1
8. Supplementary Figure *8* Empirical cumulative distribution function of DTSS - Pain domain scores by PGIS over time
9. Supplementary Figure *9* Empirical cumulative distribution function of DTSS - Extra-abdominal domain scores by PGIS over time
10. Supplementary Figure *10* Empirical cumulative distribution function of DTSS - Intra-abdominal scores by PGIC over time
11. Supplementary Figure *11* Empirical cumulative distribution function of DTIS - Physical functioning scores by PGIS over time
12. Supplementary Figure *12* Empirical cumulative distribution function of DTIS - Sleep domain scores by PGIS over time
13. Supplementary Figure *13* Empirical cumulative distribution function of DTIS - Emotional impact domain scores by PGIS over time
14. Supplementary Table *2* Convergent Validity: Correlation between DTSS total and domain scores with other measures at baseline
15. Supplementary Table *3* Convergent Validity: Correlation between DTIS domain scores with other measures at baseline
16. Supplementary Table *4* Anchor Correlations for DTSS total and domain change scores and DTIS domain change scores
17. Supplementary Table *5* Anchor Descriptive for DTSS and DTIS scores change by PGIS/PGIC at Cycles 4 and 7
18. Supplementary Table *6* Anchor Descriptive for DTSS and DTIS scores change by PGIS/PGIC at Cycles 4 and 7 (Collapsed categories)
19. Supplementary Table *7* Anchor Descriptive for DTSS and DTIS scores change between PGIS categories at Cycles 4 and 7 (uncollapsed categories)
20. Appendix 1 CFA detailed Results
21. Appendix 2 Meaningful change Modelling Approach
22. Appendix 3 Countries Included and Ethical Approval Process
23. Appendix 4 Schedule of Assessments

**Supplementary Figure 1 Boxplots of DTSS and DTIS total and domain scores by PGIS at baseline**

| 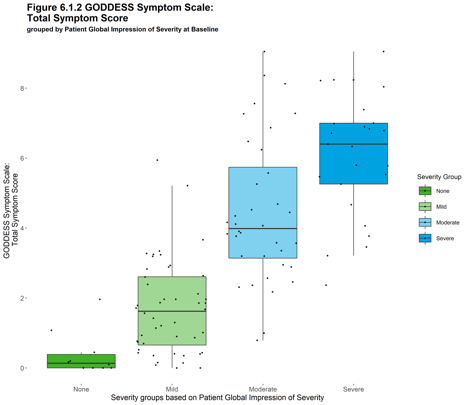 | 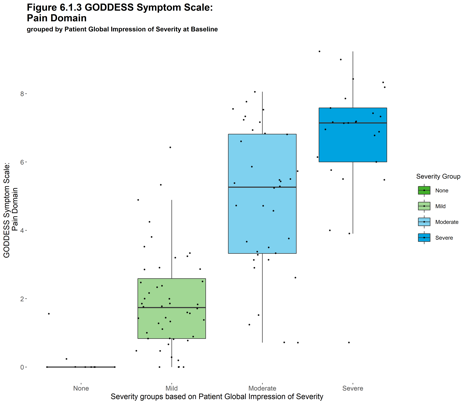 | 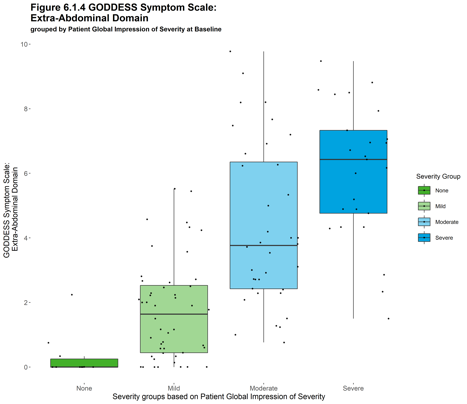 | 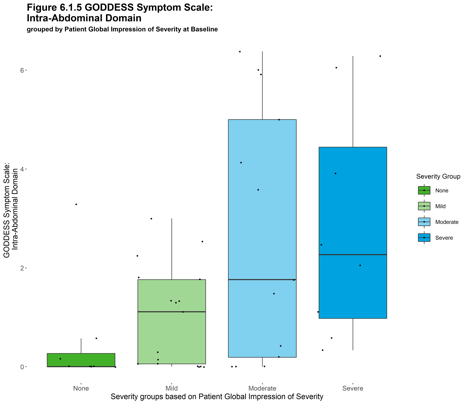 |
| --- | --- | --- | --- |
| 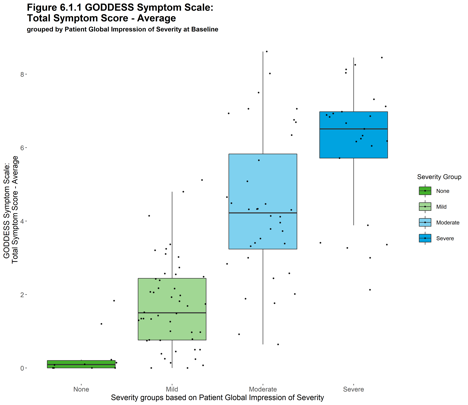 | 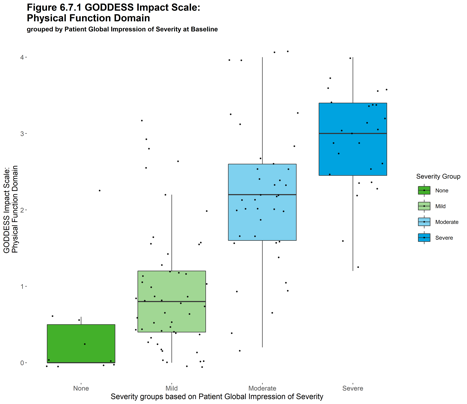 | 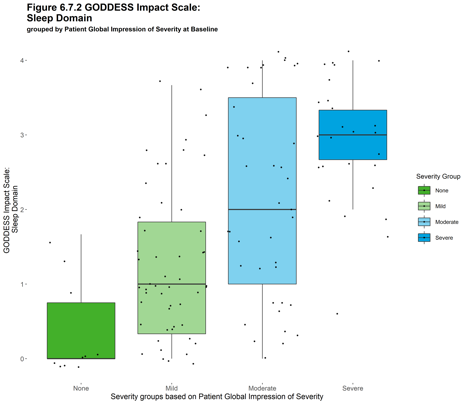 | 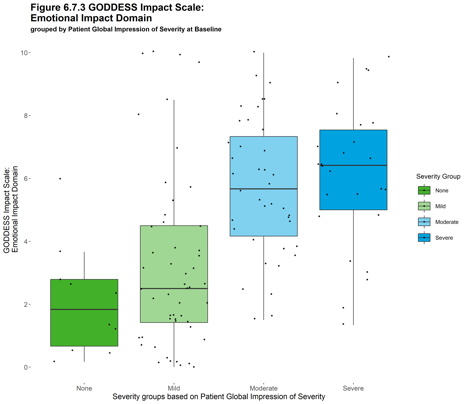 |

**Supplementary Figure 2: DTSS item distribution of daily item responses Cycle 4, Day -1**
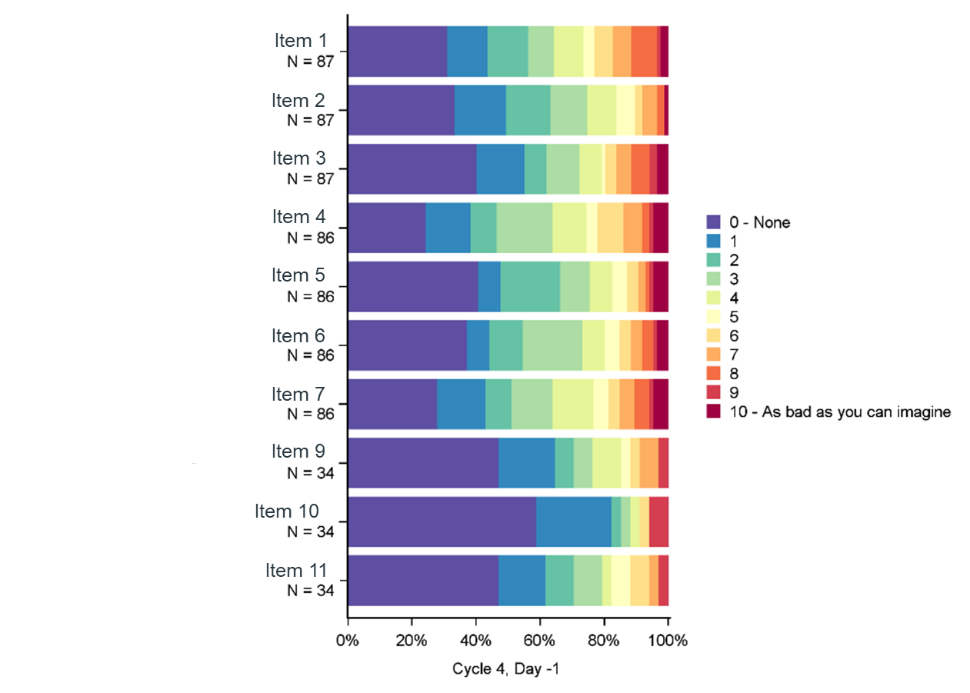


Item 1: Pain; Item 2: Dull pain; Item 3: Shooting Pain; Item 4: Fatigue; Item 5: Swelling; Item 6: Muscle weakness; Item 7: Difficulty moving; Item 9: Abdominal pain; Item 10: Nausea; Item 11: Fullness.

**Supplementary Figure 3: DTSS item distribution of daily item responses Cycle 7, Day -1**


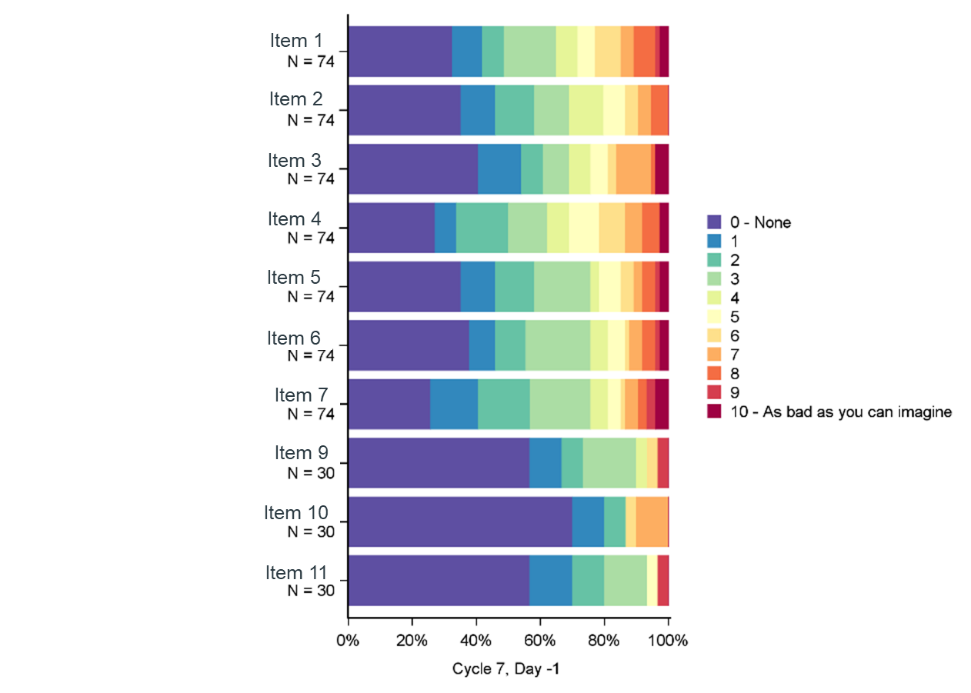


Item 1: Pain; Item 2: Dull pain; Item 3: Shooting Pain; Item 4: Fatigue; Item 5: Swelling; Item 6: Muscle weakness; Item 7: Difficulty moving; Item 9: Abdominal pain; Item 10: Nausea; Item 11: Fullness.

**Supplementary Figure 4 DTIS item distribution of daily item responses (items 1 to 9) Cycle 4, Day -1**


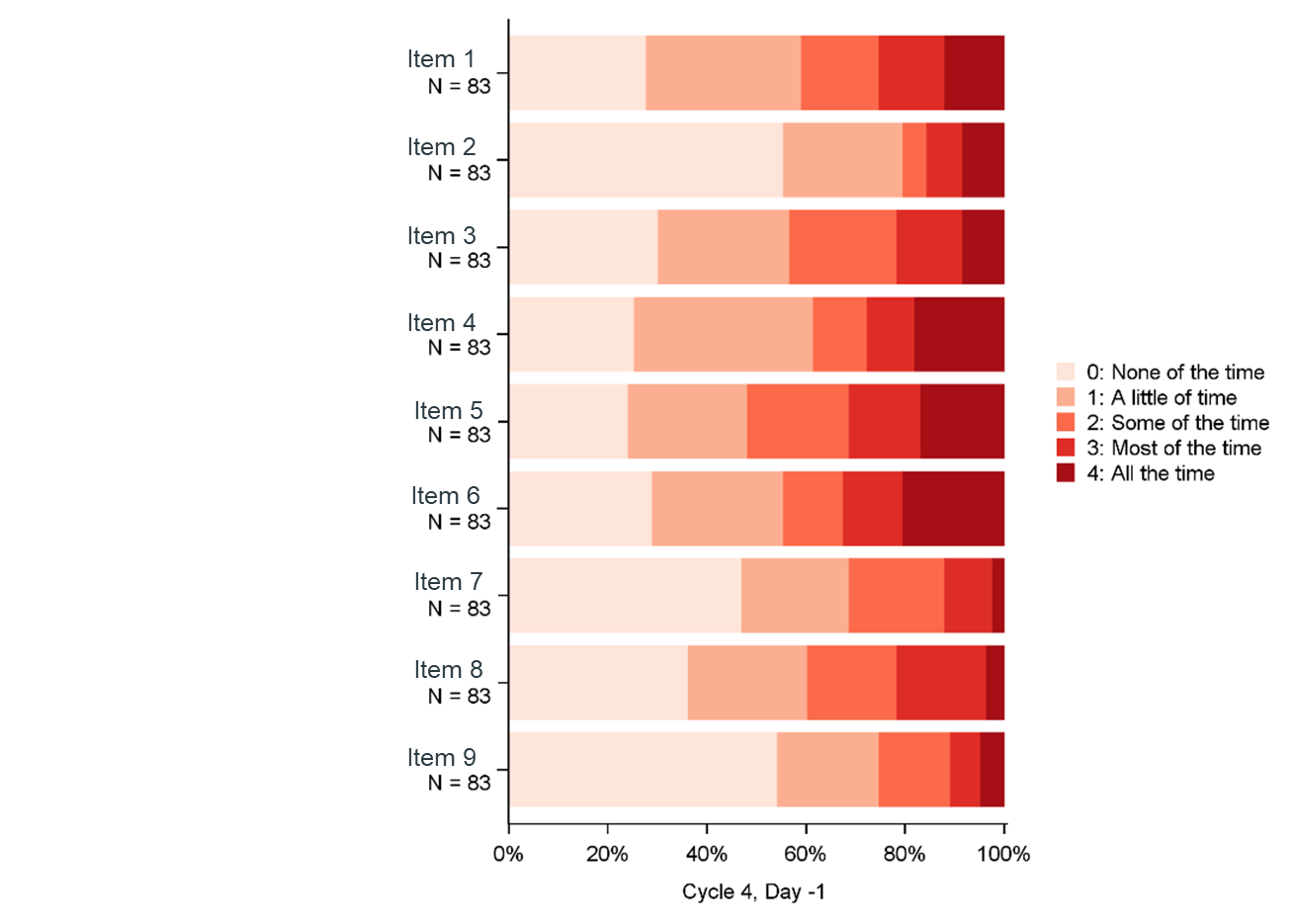


Item 1: Moving; Item 2: Reaching (frequency); Item 3: Falling asleep; Item 4: Comfortable in bed; Item 5: Staying asleep; Item 6: Vigorous activity; Item 7: Moderate activity; Item 9: Avoidance because of appearance.

**Supplementary Figure 5: DTIS item distribution of daily item (items 1 to 9) Cycle 7, Day -1**


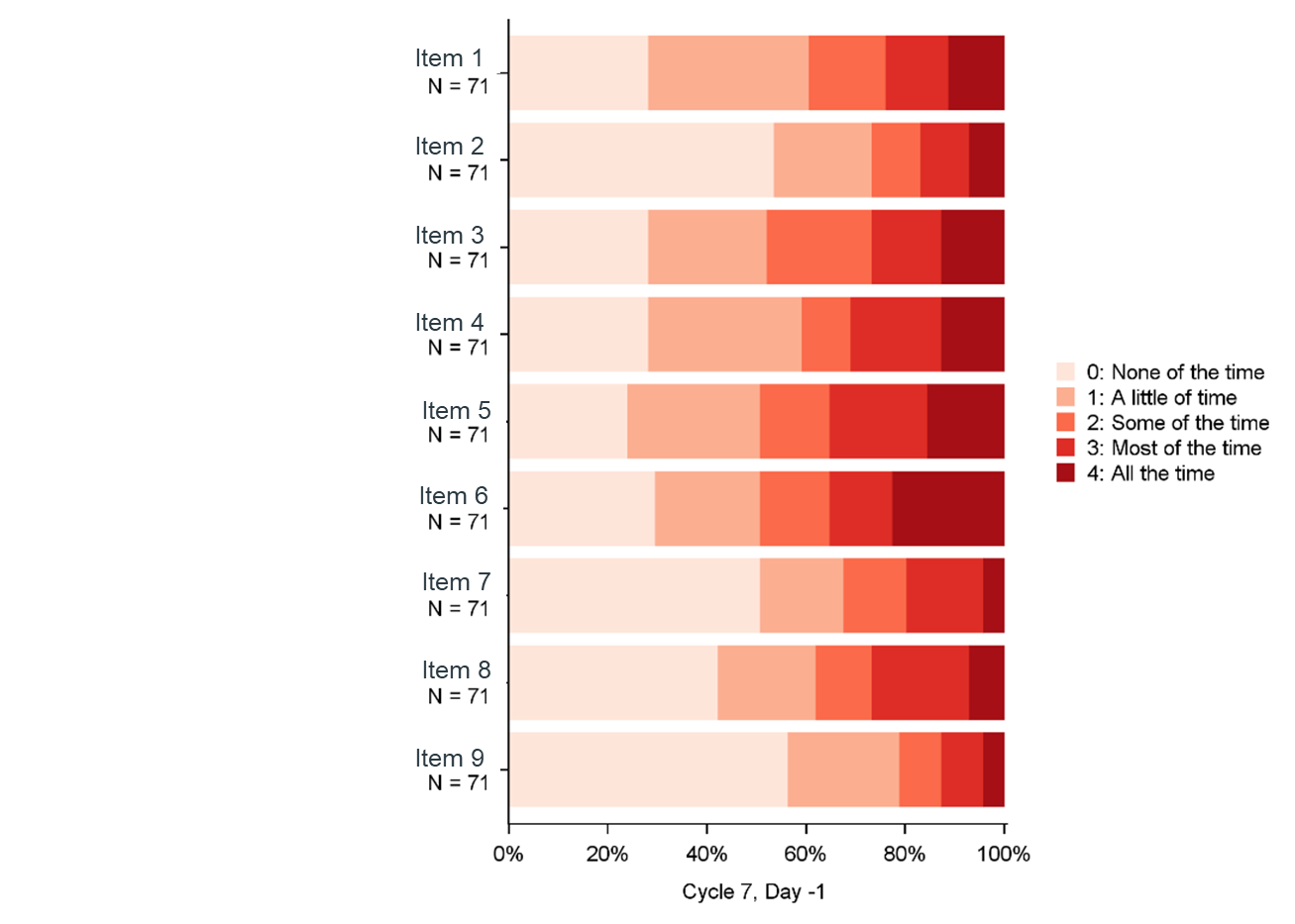


Item 1: Moving; Item 2: Reaching (frequency); Item 3: Falling asleep; Item 4: Comfortable in bed; Item 5: Staying asleep; Item 6: Vigorous activity; Item 7: Moderate activity; Item 9: Avoidance because of appearance.

**Supplementary Figure 6: DTIS item distribution of daily item responses (items 10 to 17) Cycle 4, Day -1**


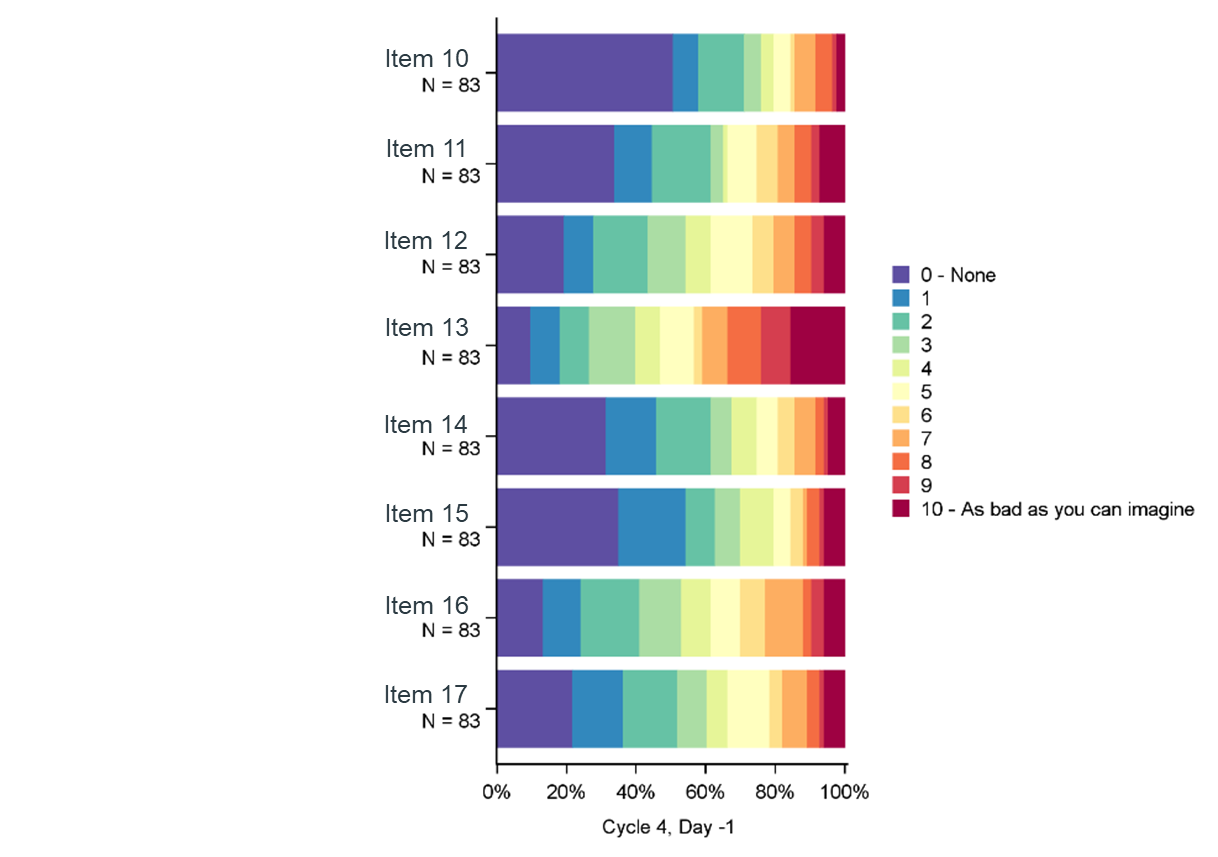


Item 10: Reaching (difficulty); Item 11: Dissatisfied with appearance; Item 12: Fear of tests; Item 13: Fear of growth/recurrence; Item 14: Hopelessness; Item 15: Anger; Item 16: Anxiety; Item 17: Frustration.

**Supplementary Figure 7: DTIS item distribution of daily item responses (items 10 to 17) Cycle 7, Day -1**


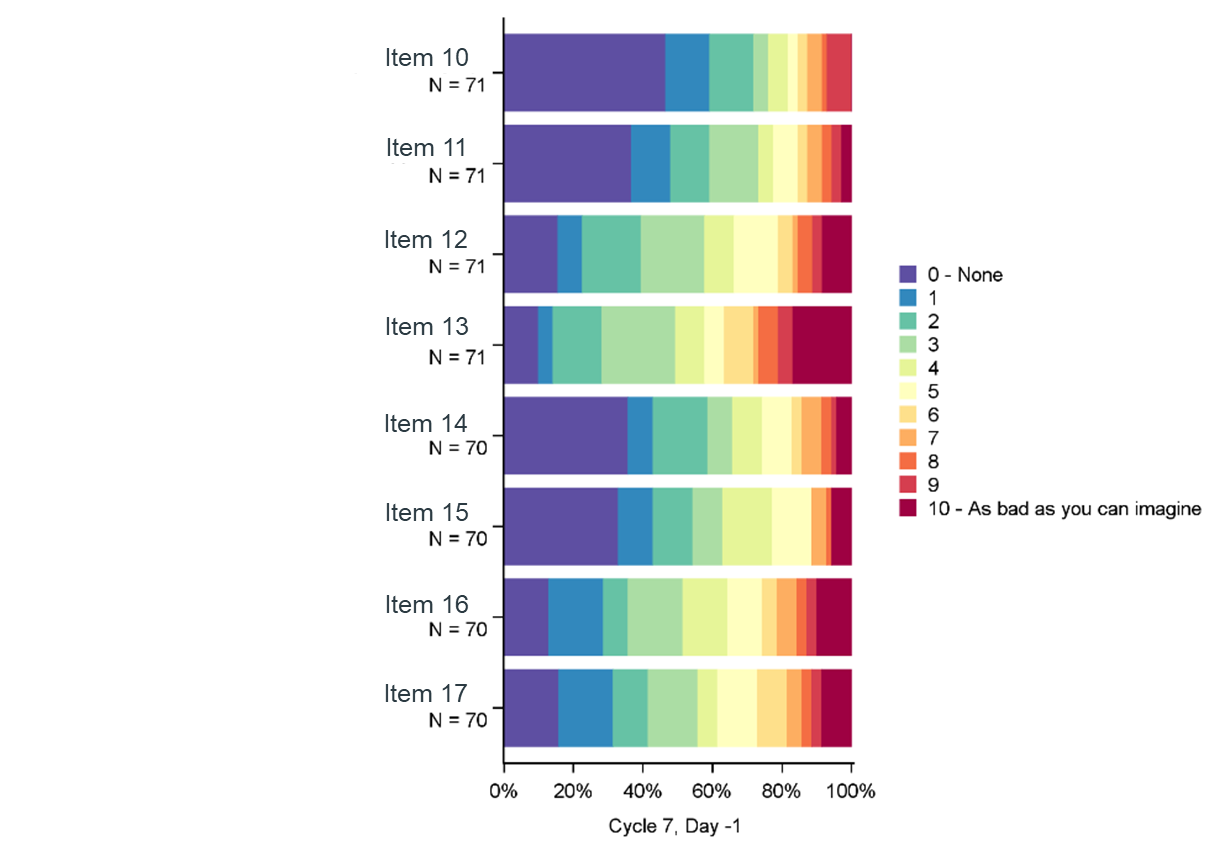


Item 10: Reaching (difficulty); Item 11: Dissatisfied with appearance; Item 12: Fear of tests; Item 13: Fear of growth/recurrence; Item 14: Hopelessness; Item 15: Anger; Item 16: Anxiety; Item 17: Frustration.

**Supplementary Figure 8 Empirical cumulative distribution function of DTSS - Pain domain scores by PGIS over time**


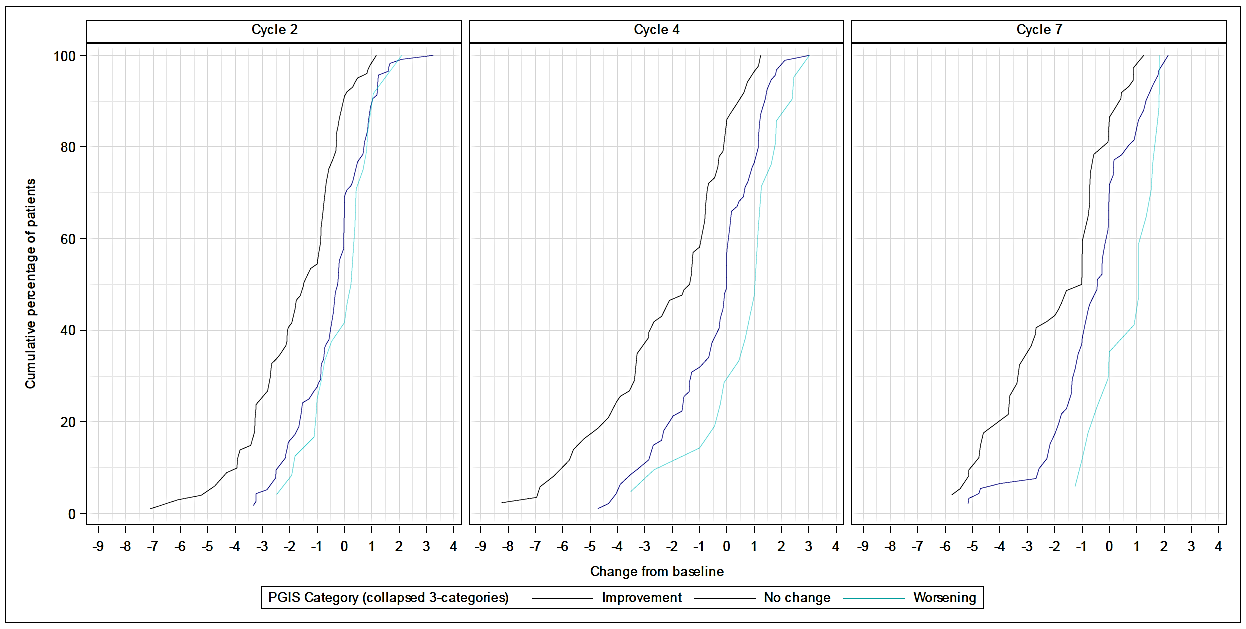


**Supplementary Figure 9 Empirical cumulative distribution function of DTSS - Extra-abdominal domain scores by PGIS over time**


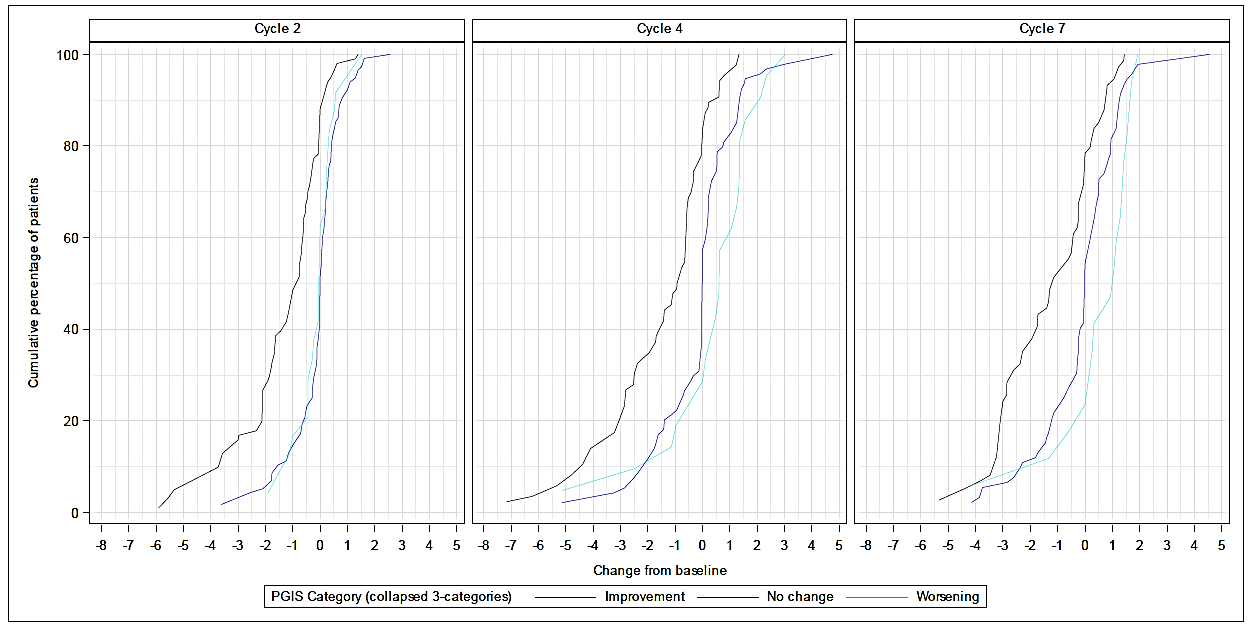


**Supplementary Figure 10 Empirical cumulative distribution function of DTSS - Intra-abdominal domain scores by PGIC over time**


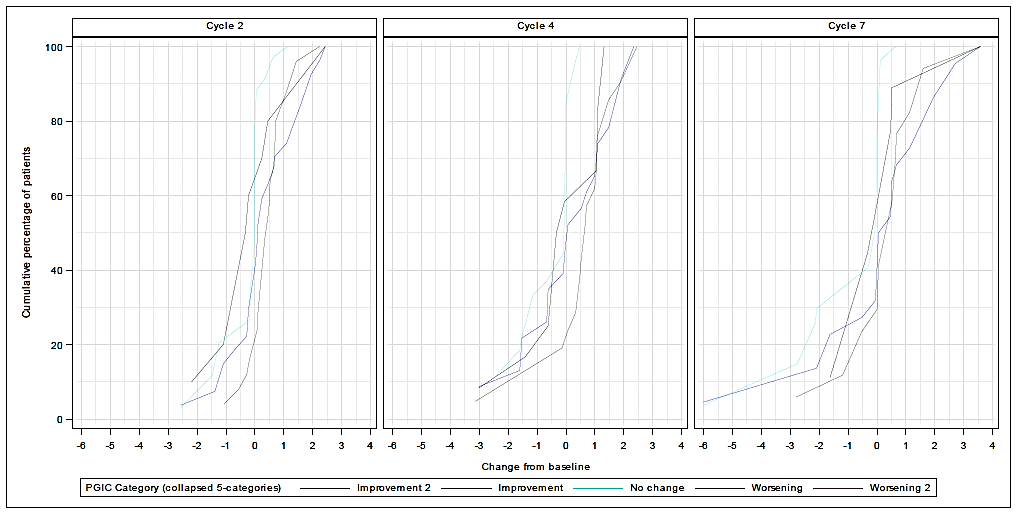


**Supplementary Figure 11 Empirical cumulative distribution function of DTIS - Physical functioning domain scores by PGIS over time**


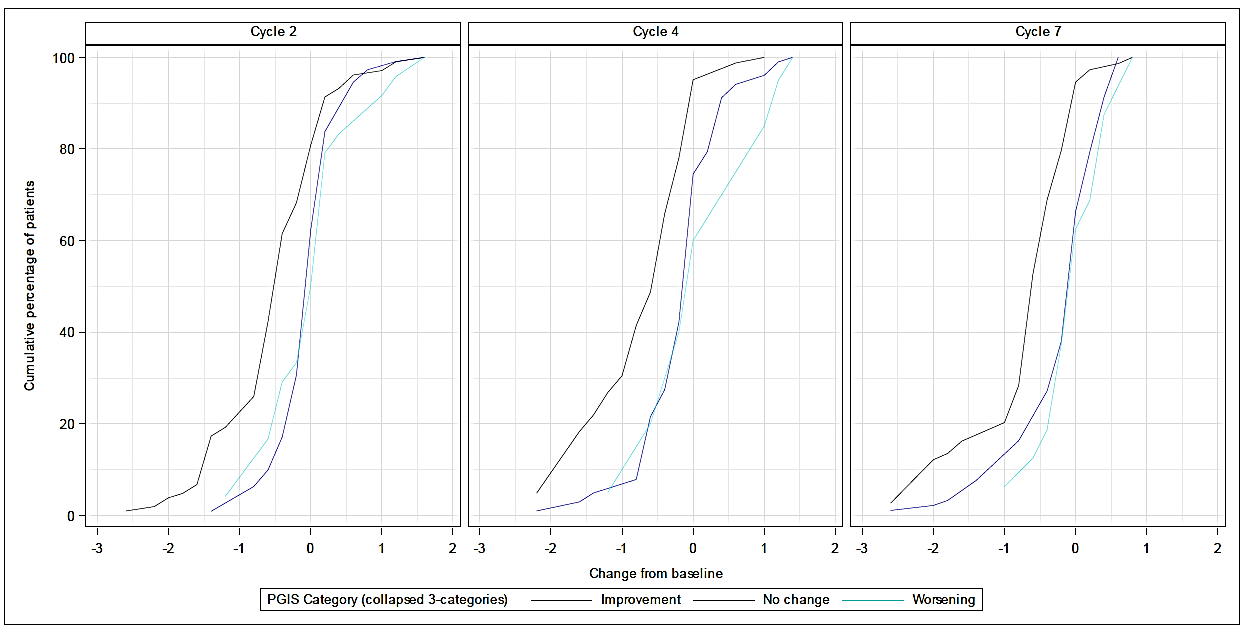


**Supplementary Figure 12 Empirical cumulative distribution function of DTIS - Sleep domain scores by PGIS over time**


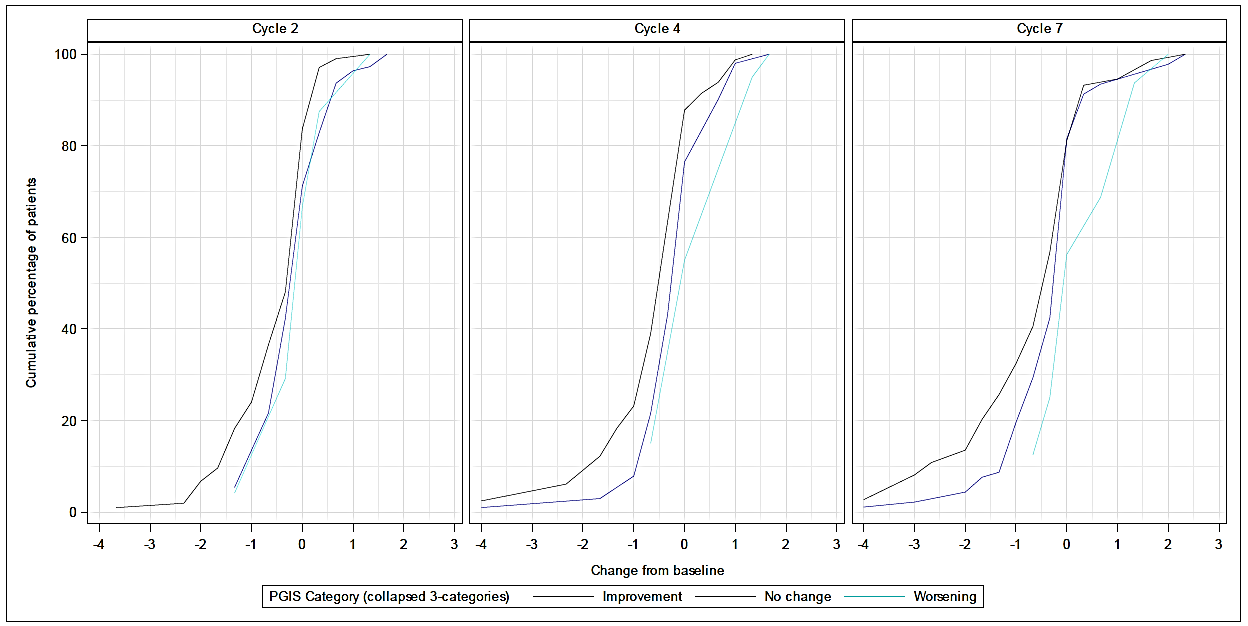


**Supplementary Figure 13 Empirical cumulative distribution function of DTIS - Emotional domain scores by PGIS over time**


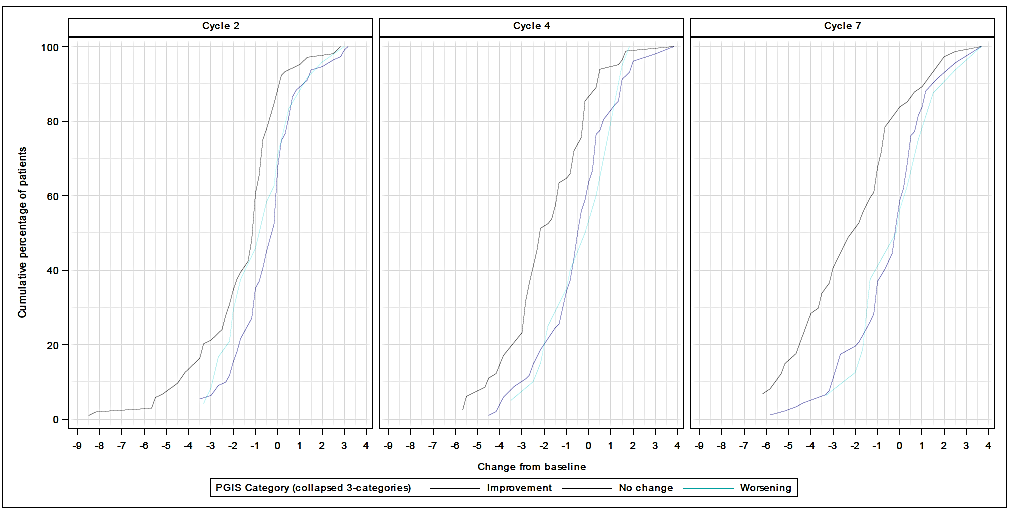


**Supplementary Table 1 DTSS and DTIS scoring**

| Scale | Domain | Number of items | Item range* | Item numbers per scale |
| --- | --- | --- | --- | --- |
| DTSS | **Pain** | 3 | 0-10 | 1-3 |
|  | **Fatigue** | 1 | 0-10 | 4 |
|  | **Extra-abdominal** | 3 | 0-10 | 5-7 |
|  | **Intra-abdominal** | 3 | 0-10 | 9-11 |
| DTIS | **Physical Functioning** | 6 | 0-4 | 1, 2, 6, 7, 8 |
|  | **Sleep** | 3 | 0-4 | 3-5 |
|  | **Emotional** | 6 | 0-10 | 12-17 |

DTSS: Desmoid Tumor Symptom Scale; DTIS: Desmoid Tumor Impact Scale. DTSS total score (daily) is calculated as the mean the following quantities: average of the 3 pain items, item 4 - item 7. DTSS total score (weekly) is calculated as the mean of the daily scores of at least 4 available days within a week.

**Supplementary Table 2 Internal Consistency: DTSS total and domain scores and DTIS domain scores**

|  |  | Cronbach Alpha (95% CI) | | |
| --- | --- | --- | --- | --- |
| Scale | **Domain** | **Baseline** | **Cycle 4** | **Cycle 7** |
| DTSS | **Total Symptom** | 0.95 (0.94; 0.96) | 0.95 (0.93; 0.96) | 0.96 (0.95; 0.98) |
|  | **Pain Domain** | 0.95 (0.93; 0.96) | 0.94 (0.91; 0.96) | 0.97 (0.96; 0.98) |
|  | **Extra-abdominal Domain** | 0.92 (0.89; 0.94) | 0.93 (0.90; 0.95) | 0.96 (0.95; 0.98) |
|  | **Intra-abdominal Domain** | 0.82 (0.71; 0.89) | 0.71 (0.49; 0.85) | 0.79 (0.62; 0.89) |
| DTIS | **Physical Functioning Domain** | 0.92 (0.89; 0.94) | 0.90 (0.87; 0.93) | 0.93 (0.90; 0.95) |
|  | **Sleep Domain** | 0.91 (0.88; 0.94) | 0.87 (0.81; 0.91) | 0.91 (0.86; 0.94) |
|  | **Emotional Domain** | 0.92 (0.90; 0.94) | 0.93 (0.90; 0.95) | 0.95 (0.93; 0.97) |

DTSS: Desmoid Tumor Symptom Scale; DTIS: Desmoid Tumor Impact Scale. For a symptom scale summary score calculated, it was assumed that at least 4 days out of 7 were available. If sufficient data was not available at Baseline, data from Screening period (if available) is used instead. CI: confidence interval.

**Supplementary Table 3 Convergent Validity: Correlation between DTSS total and domain scores with other measures at baseline**

|  | Total symptom score | Pain domain | Extra-abdominal domain | Intra-abdominal domain |
| --- | --- | --- | --- | --- |
| Score | **(N) r** | **(N) r** | **(N) r** | **(N) r** |
| BPI Pain at its Worst in Last 24 Hours | (69) 0.74 | (69) 0.87 | (69) 0.64 | (69) 0.58 |
| BPI Pain at its Least in Last 24 Hours | (69) 0.70 | (69) 0.73 | (69) 0.62 | (69) 0.68 |
| BPI Pain on the Average | (69) 0.73 | (69) 0.84 | (69) 0.64 | (69) 0.60 |
| BPI Pain Severity Subscale Score | (97) 0.80 | (97) 0.85 | (97) 0.72 | (97) 0.56 |
| PROMIS-PF short form 10a T-score | (129) -0.71 | (129) -0.65 | (129) -0.68 | (129) -0.51 |
| EORTC QLQ-C30 Global Health Score | (126) -0.55 | (126) -0.53 | (126) -0.50 | (126) -0.36 |
| EORTC QLQ-C30 Fatigue Symptom Scale | (127) 0.64 | (127) 0.64 | (127) 0.54 | (127) 0.55 |
| EORTC QLQ-C30 Nausea & Vomiting Symptom Scale | (127) 0.30 | (127) 0.28 | (127) 0.25 | (127) 0.64 |
| EORTC QLQ-C30 Pain Symptom Scale | (127) 0.71 | (127) 0.80 | (127) 0.64 | (127) 0.54 |
| EORTC QLQ-C30 Appetite Loss | (127) 0.18 | (127) 0.19 | (127) 0.13 | (127) 0.34 |

DTSS: Desmoid Tumor Symptom Scale; DTIS: Desmoid Tumor Impact Scale; BPI: Brief Pain Inventory; EORTC QLQ-C30: European Organization for research and treatment of Cancer Quality of Life Questionnaire-Core 30; PROMIS- PF: Patient Reported Outcomes Measurement Information System Physical Function; N: Number of cases. High correlation when r ≥ 0.7; moderate correlation when 0.4≤ r < 0.7 and weak correlation when 0.1 ≤ r <0.

**Supplementary Table 4 Convergent Validity: Correlation between DTIS domain scores with other measures at baseline**

|  | Physical functioning domain | Sleep domain | Emotional domain |
| --- | --- | --- | --- |
| Score | **(N) r** | **(N) r** | **(N) r** |
| BPI Interference Subscale Score | (87) 0.69 | (87) 0.51 | (87) 0.52 |
| PROMIS-PF short form Bend or Twist Your Back | (119) -0.69 | (119) -0.39 | (119) -0.38 |
| PROMIS-PF short form Reach into a High Cupboard | (119) -0.78 | (119) -0.60 | (119) -0.37 |
| PROMIS-PF short form Trouble Doing Regular Daily Work | (119) -0.88 | (119) -0.62 | (119) -0.51 |
| EORTC QLQ-C30 Physical Functioning | (127) -0.83 | (127) -0.54 | (127) -0.37 |
| EORTC QLQ-C30 Role Functioning | (127) -0.83 | (127) -0.54 | (127) -0.41 |
| EORTC QLQ-C30 Emotional Functioning | (127) -0.52 | (127) -0.54 | (127) -0.63 |
| EORTC QLQ-C30 Cognitive Functioning | (126) -0.48 | (126) -0.45 | (126) -0.28 |
| EORTC QLQ-C30 Social Functioning | (127) -0.70 | (127) -0.63 | (127) -0.52 |
| EORTC QLQ-C30 Insomnia | (127) 0.61 | (127) 0.91 | (127) 0.44 |

DTIS: Desmoid Tumor Impact Scale; BPI: Brief Pain Inventory; EORTC QLQ-C30: European Organization for research and treatment of Cancer Quality of Life Questionnaire-Core 30; PROMIS- PF: Patient Reported Outcomes Measurement Information System Physical Function; N: Number of cases. High correlation when r ≥ 0.7; moderate correlation when 0.4≤ r < 0.7 and weak correlation when 0.1 ≤ r <0.

**Supplementary Table 5 Anchor Correlations for DTSS total and domain change scores and DTIS domain change scores**

| Scale | Score | Timepoint | PGIS  (N) r | PGIC  (N) r |
| --- | --- | --- | --- | --- |
| DTSS | Total Symptom | Cycle 4 | **(73) 0.69** | **(73) -0.74** |
|  |  | Cycle 7 | **(61) 0.60** | **(61) -0.69** |
|  | Pain Domain | Cycle 4 | **(73) 0.68** | **(73) -0.74** |
|  |  | Cycle 7 | **(61) 0.58** | **(61) -0.43** |
|  | Extra-abdominal Domain | Cycle 4 | **(73) 0.64** | **(73) -0.71** |
|  |  | Cycle 7 | **(61) 0.62** | **(61) -0.71** |
|  | Intra-abdominal Domain | Cycle 4 | (28) 0.21 | **(29) -0.32** |
|  |  | Cycle 7 | (25) 0.06 | (25) -0.20 |
| DTIS | Physical Functioning Domain | Cycle 4 | **(80) 0.66** | **(79) -0.57** |
|  |  | Cycle 7 | **(67) 0.61** | **(66) -0.52** |
|  | Sleep Domain | Cycle 4 | **(80) 0.45** | **(79) -0.49** |
|  |  | Cycle 7 | **(67) 0.52** | **(66) -0.39** |
|  | Emotional Domain | Cycle 4 | **(80) 0.51** | **(79) -0.40** |
|  |  | Cycle 7 | **(67) 0.58** | **(66) -0.50** |

Polyserial correlations between change from baseline of the DTSS and DTIS scores and the change from baseline in the anchor score at the same cycle. Correlations > 0.3 considered adequate for an anchor.

PGIS: Patient Global Impression of Severity; PGIC: Patient Global Impression of Change; DTSS: Desmoid Tumor Symptom Scale; DTIS: Desmoid Tumor Impact Scale; N: number of cases.

**Supplementary Table 6 Anchor Descriptive for DTSS and DTIS scores change by PGIS/PGIC at Cycles 4 and 7 (Collapsed categories)**

|  |  | | Cycle 4 | | | | | Cycle 7 | | | | |
| --- | --- | --- | --- | --- | --- | --- | --- | --- | --- | --- | --- | --- |
| Scale | **Score** | **Category** | **N** | **Mean** | **SD** | **Median** | **95% CI** | **N** | **Mean** | **SD** | **Median** | **95% CI** |
| DTSS | Total Symptom | Improvement | 30 | **-1.64** | 1.59 | **-1.51** | [-2.24; -1.05] | 24 | **-1.32** | 1.35 | **-1.27** | [-1.89; -0.75] |
|  |  | No change | 33 | -0.09 | 0.94 | 0.00 | [-0.43; 0.24] | 32 | -0.13 | 1.45 | -0.09 | [-0.65; 0.40] |
|  |  | Worsening | 10 | 0.70 | 1.61 | 0.97 | [-0.45; 1.85] | 5 | 1.09 | 0.79 | 0.97 | [0.10; 2.07] |
|  | Pain Domain | Improvement | 30 | **-2.58** | 2.35 | **-2.24** | [-3.45; -1.70] | 24 | **-2.25** | 1.99 | **-2.00** | [-3.09; -1.41] |
|  |  | No change | 33 | -0.48 | 1.47 | -0.10 | [-1.00; 0.04] | 32 | -0.65 | 1.69 | -0.44 | [-1.26; -0.04] |
|  |  | Worsening | 10 | 1.51 | 0.78 | 1.44 | [0.95; 2.07] | 5 | 0.79 | 0.77 | 1.07 | [-0.16; 1.74] |
|  | Extra-abdominal Domain | Improvement | 30 | **-1.71** | 1.79 | **-1.25** | [-2.38; -1.04] | 24 | **-1.56** | 1.60 | **-1.56** | [-2.23; -0.88] |
|  |  | No change | 33 | -0.15 | 1.07 | 0.00 | [-0.53; 0.23] | 32 | -0.06 | 1.63 | 0.00 | [-0.65; 0.53] |
|  |  | Worsening | 10 | 0.67 | 2.32 | 1.34 | [-0.99; 2.33] | 5 | 1.27 | 0.60 | 1.42 | [0.52; 2.01] |
|  | Intra-abdominal Domain* | Improvement 2 | 6 | **0.01** | 1.63 | **0.50** | [-1.70; 1.72] | 3 | **0.21** | 0.47 | **0.48** | [ -0.96; 1.39] |
|  |  | Improvement | 7 | 0.00 | 1.14 | -0.10 | [-1.05; 1.05] | 10 | -0.39 | 2.47 | -0.03 | [ -2.15; 1.38] |
|  |  | No change | 8 | -0.59 | 1.03 | -0.03 | [-1.45; 0.27] | 8 | -0.64 | 1.14 | -0.03 | [ -1.58; 0.31] |
|  |  | Worsening | 8 | 0.91 | 0.78 | 0.69 | [0.25; 1.56] | 4 | 1.20 | 1.60 | 0.58 | [ -1.36; 3.75] |
|  |  | Worsening 2 | 0 | (NC) | (NC) | (NC) | (NC) | 0 | (NC) | (NC) | (NC) | (NC) |
| DTIS | Physical Functioning Domain | Improvement | 33 | **-0.73** | 0.65 | **-0.60** | [-0.96; -0.50] | 27 | **-0.77** | 0.73 | -**0.60** | [-1.06; -0.48] |
|  |  | No change | 37 | -0.12 | 0.51 | 0.00 | [-0.29; 0.05] | 34 | -0.11 | 0.50 | 0.00 | [-0.28; 0.07] |
|  |  | Worsening | 10 | 0.44 | 0.75 | 0.40 | [-0.09; 0.97] | 6 | 0.07 | 0.55 | 0.10 | [-0.51; 0.64] |
|  | Sleep Domain | Improvement | 33 | **-0.56** | 0.93 | -**0.33** | [-0.88; -0.23] | 27 | **-0.84** | 1.27 | -**0.33** | [-1.34; -0.34] |
|  |  | No change | 37 | -0.05 | 0.65 | 0.00 | [-0.26; 0.17] | 34 | -0.23 | 0.75 | 0.00 | [-0.49; 0.03] |
|  |  | Worsening | 10 | 0.40 | 0.81 | 0.50 | [-0.18; 0.98] | 6 | 0.67 | 0.97 | 0.67 | [-0.35; 1.68] |
|  | Emotional Domain | Improvement | 33 | **-1.90** | 1.89 | -**2.17** | [-2.57; -1.23] | 27 | **-2.73** | 2.22 | -**3.00** | [-3.61; -1.86] |
|  |  | No change | 37 | -0.25 | 1.67 | -0.17 | [-0.80; 0.31] | 34 | -0.16 | 1.50 | 0.00 | [-0.68; 0.36] |
|  |  | Worsening | 10 | -0.05 | 1.49 | 0.08 | [-1.11; 1.01] | 6 | 0.50 | 2.28 | -0.08 | [-1.89; 2.89] |

DTSS: Desmoid Tumor Symptom Scale; DTIS: Desmoid Tumor Impact Scale. For a symptom scale summary score calculation, it was assumed that at least 4 days out of 7 were available. If sufficient data was not available at baseline, data from the screening period (if available) was used instead. PGIS collapsed categories (i.e.: Improvement, No change, and Worsening) were based on the change score from baseline at the associated later timepoint: Improvement was defined as an improve of at least one value, No change defined as same value as baseline, and Worsening was defined as a worsen of at least one value. PGIC collapsed categories (i.e.: Improvement2, Improvement, No change, Worsening, and Worsening 2) were based on the actual score at the associated later timepoint: Improvement 2 was defined as ‘Very much improved’ score, Improvement defined as ‘Much improved’ and ‘Minimally improved’ scores, No change defined as ‘No change’ score, and Worsening was defined as ‘Minimally worse’ and ‘Much worse’ scores, and Worsening2 as ‘Very much worse’ score.

*For Intra-abdominal domain scores, the anchor reported was PGIC.

**Supplementary Table 7 Anchor Descriptive for DTSS and DTIS scores change between PGIS categories at Cycles 4 and 7 (uncollapsed categories)**

|  |  |  | Cycle 4 | | | Cycle 7 | | |
| --- | --- | --- | --- | --- | --- | --- | --- | --- |
| Scale | **Score** | **Category** | **N** | **Mean** | **95% CI** | **N** | **Mean** | **95% CI** |
| DTSS | Total Symptom | Improved 3 categories | 0 | (NC) | (NC) | 1 | -4.25 | (NC) |
|  |  | Improved 2 categories | 8 | -2.99 | [ -4.52; -1.45] | 6 | -1.94 | [ -2.85; -1.02] |
|  |  | Improved 1 category | 22 | -1.16 | [ -1.69; -0.62] | 17 | **-0.93** | [ -1.57; -0.29] |
|  |  | No change | 33 | -0.09 | [ -0.43; 0.24] | 32 | **-0.13** | [ -0.65; 0.40] |
|  |  | Worsened 1 category | 10 | 0.70 | [ -0.45; 1.85] | 5 | 1.09 | [ 0.10; 2.07] |
|  |  | Worsened 2 categories | 0 | (NC) | (NC) | 0 | (NC) | (NC) |
|  |  | Worsened 3 categories | 0 | (NC) | (NC) | 0 | (NC) | (NC) |
|  | Pain Domain | Improved 3 categories | 0 | (NC) | (NC) | 1 | -5.15 | (NC) |
|  |  | Improved 2 categories | 8 | -3.68 | [ -5.34; -2.02] | 6 | -3.01 | [ -4.94; -1.07] |
|  |  | Improved 1 category | 22 | -2.17 | [ -3.23; -1.12] | 17 | **-1.81** | [ -2.80; -0.82] |
|  |  | No change | 33 | -0.48 | [ -1.00; 0.04] | 32 | **-0.65** | [ -1.26; -0.04] |
|  |  | Worsened 1 category | 10 | 1.51 | [ 0.95; 2.07] | 5 | 0.79 | [ -0.16; 1.74] |
|  |  | Worsened 2 categories | 0 | (NC) | (NC) | 0 | (NC) | (NC) |
|  |  | Worsened 3 categories | 0 | (NC) | (NC) | 0 | (NC) | (NC) |
|  | Extra-abdominal Domain | Improved 3 categories | 0 | (NC) | (NC) | 1 | -4.32 | (NC) |
|  |  | Improved 2 categories | 8 | -3.50 | [ -5.19; -1.80] | 6 | -2.51 | [ -3.15; -1.87] |
|  |  | Improved 1 category | 22 | -1.07 | [ -1.60; -0.54] | 17 | **-1.06** | [ -1.86; -0.25] |
|  |  | No change | 33 | -0.15 | [ -0.53; 0.23] | 32 | **-0.06** | [ -0.65; 0.53] |
|  |  | Worsened 1 category | 10 | 0.67 | [ -0.99; 2.33] | 5 | 1.27 | [ 0.52; 2.01] |
|  |  | Worsened 2 categories | 0 | (NC) | (NC) | 0 | (NC) | (NC) |
|  |  | Worsened 3 categories | 0 | (NC) | (NC) | 0 | (NC) | (NC) |
|  | Intra-abdominal Domain* | Very much improved | 6 | 0.01 | [-1.70; 1.72] | 3 | 0.21 | [ -0.96; 1.39] |
|  |  | Much improved | 0 | (NC) | (NC) | 7 | -1.22 | [ -3.44; 1.01] |
|  |  | Minimally improved | 7 | 0.00 | [ -1.05; 1.05] | 3 | **1.55** | [ -1.92; 5.02] |
|  |  | No change | 8 | -0.59 | [ -1.45; 0.27] | 8 | **-0.64** | [ -1.58; 0.31] |
|  |  | Minimally worse | 5 | 0.45 | [ 0.02; 0.87] | 3 | 1.38 | [ -3.37; 6.13] |
|  |  | Much worse | 3 | 1.67 | [ -0.06; 3.41] | 1 | 0.64 | (NC) |
|  |  | Very much worse | 0 | (NC) | (NC) | 0 | (NC) | (NC) |
| DTIS | Physical Functioning Domain | Improved 3 categories | 0 | (NC) | (NC) | 1 | -1.60 | (NC) |
|  |  | Improved 2 categories | 8 | -1.23 | [ -1.76; -0.69] | 7 | -1.34 | [ -2.14; -0.55] |
|  |  | Improved 1 category | 25 | -0.57 | [ -0.80; -0.33] | 19 | **-0.52** | [ -0.77; -0.26] |
|  |  | No change | 37 | -0.12 | [ -0.29; 0.05] | 34 | **-0.11** | [ -0.28; 0.07] |
|  |  | Worsened 1 category | 10 | 0.44 | [ -0.09; 0.97] | 6 | 0.07 | [ -0.51; 0.64] |
|  |  | Worsened 2 categories | 0 | (NC) | (NC) | 0 | (NC) | (NC) |
|  |  | Worsened 3 categories | 0 | (NC) | (NC) | 0 | (NC) | (NC) |
|  | Sleep Domain | Improved 3 categories | 0 | (NC) | (NC) | 1 | -2.00 | (NC) |
|  |  | Improved 2 categories | 8 | -0.79 | [ -2.03; 0.45] | 7 | -1.71 | [ -3.12; -0.31] |
|  |  | Improved 1 category | 25 | -0.48 | [ -0.77; -0.19] | 19 | **-0.46** | [ -0.94; 0.03] |
|  |  | No change | 37 | -0.05 | [ -0.26; 0.17] | 34 | **-0.23** | [ -0.49; 0.03] |
|  |  | Worsened 1 category | 10 | 0.40 | [ -0.18; 0.98] | 6 | 0.67 | [-0.35; 1.68] |
|  |  | Worsened 2 categories | 0 | (NC) | (NC) | 0 | (NC) | (NC) |
|  |  | Worsened 3 categories | 0 | (NC) | (NC) | 0 | (NC) | (NC) |
|  | Emotional Domain | Improved 3 categories | 0 | (NC) | (NC) | 1 | -0.67 | (NC) |
|  |  | Improved 2 categories | 8 | -3.35 | [ -4.48; -2.22] | 7 | -3.83 | [ -6.50; -1.17] |
|  |  | Improved 1 category | 25 | -1.44 | [ -2.19; -0.69] | 19 | **-2.44** | [ -3.35; -1.53] |
|  |  | No change | 37 | -0.25 | [ -0.80; 0.31] | 34 | **-0.16** | [ -0.68; 0.36] |
|  |  | Worsened 1 category | 10 | -0.05 | [ -1.11; 1.01] | 6 | 0.50 | [ -1.89; 2.89] |
|  |  | Worsened 2 categories | 0 | (NC) | (NC) | 0 | (NC) | (NC) |
|  |  | Worsened 3 categories | 0 | (NC) | (NC) | 0 | (NC) | (NC) |

DTSS: Desmoid Tumor Symptom Scale; DTIS: Desmoid Tumor Impact Scale. For a symptom scale summary score calculation, it was assumed that at least 4 days out of 7 were available. If sufficient data was not available at baseline, data from the screening period (if available) was used instead.

*For Intra-abdominal domain scores, the anchor reported was PGIC.

**Appendix 1.**

A set of confirmatory factor analyses (CFA) was carried out for the items of the GODDESS symptom and impact scales (i.e., DTSS and DTIS, respectively). The analysis was performed on the baseline Intent-to-Treat population. For the DTSS only (which contains multiple diary days), longitudinal CFA was conducted at baseline incorporating up to 7 days of data for each participant.

CFA is a technique that confirms the factor structure of a set of items. This means it verifies whether the data can support that all the items fit a pre-specified model that can be either unidimensional (i.e., all items create a single scale) or multi-dimensional (i.e., items form various domains or scales).

For the DTSS and DTIS, full information maximum likelihood (FIML) method was used to account for potential missing items within the baseline data and maximize the data used. This method assumes input data are multivariate normal, which may be approximately true for ordinal data with five or more categories (Norman, 2010); (Zumbo & Zimmerman, 1993).

Models were judged on their approximate fit indices: Comparative Fit Index (CFI) >0.95, Tucker-Lewis Index (TLI) >0.90, Root Mean Square Error of Approximation (RMSEA) <0.06, and Standardized Root Mean Residual (SRMR) <0.08 (Hu & Bentler, 1999; Brown, 2015). To obtain finer factor solutions, magnitude of factor loadings, modification indices, and standardized residuals were also assessed. Their magnitude was evaluated in comparison to other items in the scale and in an integrative manner, taking all three under consideration at once, so no strict thresholds were applied for each criterion. Where modification indices and residual variances were assessed, values >100 and correlations > |0.10|, respectively, were taken as indices of lack of fit (local dependence), previous literature has also suggested that smaller values could lead to revisions in the model if multiple modification indices or
residual variances consistently showed the same pattern of results (Watt, et al., 2014). In the presence of local correlations and to improve model fit, local dependence between items may be introduced where the latent variables are not sufficient to explain the association between items. Introduction of local correlations was based on both modification indices and content assessment. A final model was selected based on improvement in goodness-of-fit and localized areas of ill fit (modification indices, standardized residuals), in tandem with prior qualitative research and strong theory.

- *Brown AT (2015). Confirmatory Factor Analysis for Applied Research. New York: The Guilford Press 2015*
- *Hu L and Bentler P (1999). Cutoff criteria for fit indexes in covariance structure analysis: Conventional criteria versus new alternatives, Structural Equation Modeling 1999 (Vol. 6:1). doi:* [*https://doi.org/10.1080/10705519909540118*](https://doi.org/10.1080/10705519909540118)
- Norman, G. (2010). Likert scales, levels of measurement and the “laws” of statistics. Advances in health sciences education, 15, 625-632.
- Zumbo, B. D., & Zimmerman, D. W. (1993). Is the selection of statistical methods governed by level of measurement?. Canadian Psychology/Psychologie canadienne, 34(4), 390.
- Watt, T., Groenvold, M., Deng, N., Gandek, B., Feldt-Rasmussen, U., Rasmussen, Å. K., ... & Bjorner, J. B. (2014). Confirmatory factor analysis of the thyroid-related quality of life questionnaire ThyPRO. Health and Quality of Life Outcomes, 12, 1-12

**GODDESS PRO Symptom Scale: DTSS**

Different score models were tested to confirm the factor structure of GODDESS PRO symptom items as well as potential underlying subdomains. This analysis aimed to use the available data in the context of a daily diary study in a longitudinal CFA to increase the planned analysis power and differentiate the within- and between-subjects variability.

Unidimensional models (including all items) had poor fit (**Appendix Table 1**). This was likely due to the items relating to intra-abdominal domain only being appropriate for a subset of patients with desmoid tumour. The second, unidimensional model tested, which excluded the intra-abdominal items, had better fit overall, but still fell short of the fit indices indicative of an appropriate model. To assess whether this was because of unaccounted inter-relationships among some of the items, two bi-factor models were developed. One model assessed the pain items (item 1-3) as a domain (while other items loaded onto the general factor only). The second bi-factor model also assessed the extra-abdominal symptoms (item 5-7) as its own domain and allowed the fatigue item (item 4) to load only onto the general factor. These 2 models had appropriate fit, suggesting that there may be a total symptom score among these 7 items, but that “pain” and “extra-abdominal” domains also exist.

The factor loadings of the final bi-factor model were assessed to understand best how to create the total symptom score (**Appendix Table 2**). Residual correlations showed no remaining association (i.e., <0.030) among the items after accounting for the model (**Appendix Table 3**).

Although a strict factor weighting may not always be appropriate, due to factor indeterminacy issues, sometimes the loadings along with theory can inform some more nuanced scoring algorithm. The loadings showed that the pain items (which were all highly related to one another in the inter-item correlation assessment) had substantially higher loadings than the other items on the general factor. This was considered when deriving the scoring algorithm. Hence it was concluded that the DTSS total score would be based on items 1-7 but firstly by considering the average of items 1-3 to account for the over presentation of pain items. In addition, a DTSS pain domain, a DTSS intra-abdominal domain, and a DTSS extra-abdominal domain emerged, and scores were also calculated for them.

**Appendix Table 1 Model fit statistics for CFA- DTSS**

| Fit statistics | Model | | | |
| --- | --- | --- | --- | --- |
|  | **Unidimensional Model** | **Unidimensional Model (items 1-7)** | **Bi-factor Model Version 1 (One Specific Domain)** | **Bi-factor Model Version 2 (Two Specific Domains)** |
| **Model chi square** | 301.73 | 197.7 | 50.05 | 19.03 |
| **Model chi square DF** | 70 | 28 | 22 | 16 |
| **Baseline chi square** | 1569.66 | 1202.46 | 1202.46 | 1202.46 |
| **Baseline chi square DF** | 90 | 42 | 42 | 42 |
| **CFI** | 0.84 | 0.85 | 0.98 | 1.00 |
| **TLI** | 0.80 | 0.78 | 0.95 | 0.99 |
| **RMSEA estimate** | 0.06 | 0.09 | 0.04 | 0.02 |
| **SRMR** | 0.09 | 0.06 | 0.03 | 0.01 |

CFA: Confirmatory Factor Analysis; DTSS: Desmoid Tumor Symptom Scale; CFI: Comparative fit index; DF: Degrees of freedom; RMSEA: Root Mean Square Error of Approximation; SRMR: Standardized Root Mean Residual; TLI: Tucker Lewis Index.

**Appendix Table 2 Factor Loadings for Bi-factor model version 2 - DTSS**

| Item | General Factor Loading | General Factor SE | Specific Factor Loading | Specific Factor SE |
| --- | --- | --- | --- | --- |
| Item 01 Pain | 0.92 | 0.02 | 0.34 | 0.11 |
| Item 02 Dull Pain | 0.90 | 0.03 | 0.64 | 0.10 |
| Item 03 Shooting Pain | 0.89 | 0.03 | 0.33 | 0.09 |
| Item 04 Fatigue | 0.82 | 0.03 | NA | NA |
| Item 05 Swelling | 0.73 | 0.05 | 0.35 | 0.09 |
| Item 06 Muscle Weakness | 0.78 | 0.05 | 0.16 | 0.12 |
| Item 07 Difficulty Moving | 0.86 | 0.04 | 0.36 | 0.10 |

NA: Not assessed; SE: Standard error.

**Appendix Table 3 Item residual correlation matrix: Bi-factor model version 2 -DTSS**

| Item | Item 1 | Item 2 | Item 3 | Item 4 | Item 5 | Item 6 |
| --- | --- | --- | --- | --- | --- | --- |
| Item 01 Pain | 0.00 |  |  |  |  |  |
| Item 03 Dull pain | 0.00 | 0 |  |  |  |  |
| Item 04 Shooting Pain | -0.02 | 0.03 | -0.03 |  |  |  |
| Item 05 Swelling | -0.01 | 0.01 | 0.00 | -0.01 |  |  |
| Item 06 Muscle Weakness | 0.01 | -0.01 | 0.02 | -0.00 | 0.00 |  |
| Item 07 Difficulty moving | 0.02 | -0.03 | 0.03 | 0.00 | 0.00 | 0.00 |

DTSS: Desmoid Tumor Symptom Scale

**GODDESS PRO Impact Scale: DTIS**

As above, several models were assessed to understand whether the GODDESS PRO impact scale (i.e., DTIS) was appropriate to form a total overall score as well as subdomain scores. These models initially tested the presence of a unidimensional structure. The overall unidimensional model including all items showed poor fit, and this is somewhat expected given the multi-dimensional nature of the items. Based on items correlation and clinical impact regarding the conceptualisation of this tool, two bi-factor models including a general factor and subdomain factor that try to capture further explained variability were investigated. Bi-factor model version 1 was comprised by all items 1-17 that loaded on the general factor and 2 subdomain factors: items 1-11 loaded on a subdomain factor called ‘physical impact’ and items 12-17 loaded on another subdomain factor called ‘emotional impact’. Bi-factor model version 2 was comprised of all items 1-17 that loaded on the general factor and 2 subdomain factors: items 1-9 loaded on a subdomain factor called ‘physical impact’ and items 12-17 loaded on the other subdomain factor called ‘emotional impact’. The two bi-factor models showed appropriate fit, however, factor loadings in the general factor were low and this was evidence that there was limited support for an overall general factor. As such and aiming to provide a final score for the DTSS, a 3-factor correlated model was investigated as developed from 3 correlated factors: Physical functioning (items 1, 2, 6, 7, 8, 10), Emotional impact (items 12-17), and Sleep (items 3-5). This model showed good fit. Factor loadings for the correlated factors model showed high and relatively equal loadings for each of the items on their respective domains (**Appendix Table 5**). Residual correlations showed that the domains accounted for the shared variance between the items, and no residual variance remained unaccounted for (**Appendix Table 6**). Considering all these, 3 domain scores were suggested for the DTIS: physical functioning, sleep, and emotional.

**Appendix Table 4 Model fit statistics - DTIS**

| Fit statistics | Model | | | |  |
| --- | --- | --- | --- | --- | --- |
|  | **Unidimensional Model** | **Unidimensional Model (items 1-8)** | **Bi-factor Model Version 1** | **Bi-factor Model Version 2 (Exclude Items 10 and 11)** | **Correlated factors model** |
| **Model chi square** | 479.10 | 157.62 | 199.07 | 197.34 | 180.67 |
| **Model chi square DF** | 119 | 20 | 102 | 104 | 87 |
| **Baseline chi square** | 2769.39 | 3887.59 | 2769.39 | 2769.39 | 2660.62 |
| **Baseline chi square DF** | 136 | 28 | 136 | 136 | 105 |
| **CFI** | 0.86 | 0.96 | 0.96 | 0.97 | 0.96 |
| **TLI** | 0.84 | 0.95 | 0.95 | 0.95 | 0.96 |
| **RMSEA estimate** | 0.15 | 0.23 | 0.09 | 0.08 | 0.09 |
| **SRMR** | 0.14 | 0.09 | 0.06 | 0.06 | 0.05 |

DTIS: Desmoid Tumor Impact Scale; CFI: Comparative fit index; DF: Degrees of freedom; RMSEA: Root Mean Square Error of Approximation; SRMR: Standardized Root Mean Residual; TLI: Tucker Lewis Index.

**Appendix Table 5 Factor Loadings for the 3-factors correlated - DTIS**

| Item | Domain Factor loading | Domain Factor SE |
| --- | --- | --- |
| Item 01 Moving | 0.81 | 0.03 |
| Item 02 Reaching (Freq) | 0.86 | 0.03 |
| Item 06 Vigorous Activity | 0.91 | 0.02 |
| Item 07 Moderate Activity | 0.95 | 0.01 |
| Item 08 Accomplished Less | 0.88 | 0.02 |
| Item 10 Reaching (Severity) | 0.76 | 0.05 |
| Item 03 Falling Asleep | 0.94 | 0.01 |
| Item 04 Comfortable in Bed | 0.95 | 0.01 |
| Item 05 Staying Asleep | 0.83 | 0.03 |
| Item 12 Fear of Tests | 0.71 | 0.06 |
| Item 13 Fear of Growth/Recurrence | 0.78 | 0.06 |
| Item 14 Hopelessness | 0.86 | 0.04 |
| Item 15 Anger | 0.73 | 0.06 |
| Item 16 Anxiety | 0.80 | 0.05 |
| Item 17 Frustration | 0.95 | 0.05 |
| Physical Function with Emotion | 0.77 | 0.04 |
| Physical Function with Sleep | 0.51 | 0.06 |
| Emotion with Sleep | 0.55 | 0.07 |

DTIS: Desmoid Tumor Impact Scale; SE: Standard error.

**Appendix Table 6 Item residual correlation matrix: the 3-factors correlated - DTIS**

| Item | Item 1 | Item 2 | Item 6 | Item 7 | Item 8 | Item 10 | Item 3 | Item 4 | Item 5 | Item 12 | Item 13 | Item 14 | Item 15 | Item 16 |
| --- | --- | --- | --- | --- | --- | --- | --- | --- | --- | --- | --- | --- | --- | --- |
| Item 01 Moving |  |  |  |  |  |  |  |  |  |  |  |  |  |  |
| Item 02 Reaching (Freq) | -0.05 |  |  |  |  |  |  |  |  |  |  |  |  |  |
| Item 06 Vigorous Activity | -0.03 | 0.10 |  |  |  |  |  |  |  |  |  |  |  |  |
| Item 07 Moderate Activity | 0.11 | 0.08 | -0.03 |  |  |  |  |  |  |  |  |  |  |  |
| Item 08 Accomplished Less | -0.08 | 0.01 | 0.05 | -0.00 |  |  |  |  |  |  |  |  |  |  |
| Item 10 Reaching (Severity) | 0.02 | -0.12 | -0.10 | -0.01 | -0.11 |  |  |  |  |  |  |  |  |  |
| Item 03 Falling Asleep | 0.00 | -0.09 | -0.04 | 0.02 | -0.08 | 0.02 |  |  |  |  |  |  |  |  |
| Item 04 Comfortable in Bed | -0.07 | 0.01 | -0.08 | -0.07 | -0.09 | 0.02 | 0.03 |  |  |  |  |  |  |  |
| Item 05 Staying Asleep | -0.05 | 0.24 | 0.04 | 0.03 | -0.02 | -0.14 | -0.14 | -0.04 |  |  |  |  |  |  |
| Item 12 Fear of Tests | 0.02 | 0.07 | -0.05 | -0.01 | -0.05 | 0.00 | -0.07 | 0.02 | 0.09 |  |  |  |  |  |
| Item 13 Fear of Growth/Recurrence | -0.01 | -0.03 | -0.02 | 0.05 | 0.01 | 0.03 | -0.02 | 0.04 | -0.03 | 0.15 |  |  |  |  |
| Item 14 Hopelessness | 0.05 | -0.01 | 0.00 | 0.01 | -0.06 | 0.02 | -0.08 | 0.04 | 0.03 | 0.02 | -0.02 |  |  |  |
| Item 15 Anger | -0.02 | -0.00 | 0.08 | 0.01 | -0.03 | -0.06 | -0.12 | 0.01 | 0.01 | -0.01 | 0.02 | 0.13 |  |  |
| Item 16 Anxiety | -0.04 | 0.05 | 0.01 | -0.02 | 0.00 | -0.05 | -0.09 | 0.03 | 0.04 | 0.11 | 0.04 | -0.00 | 0.06 |  |
| Item 17 Frustration | 0.04 | 0.06 | 0.04 | 0.02 | 0.02 | 0.03 | -0.04 | 0.04 | 0.06 | -0.13 | -0.14 | -0.07 | 0.06 | -0.06 |

DTIS: Desmoid Tumor Impact Scale.

**Appendix 2.**

**Modeling Approach**

To understand the meaningful change thresholds in the face of missing data, a modeling approach was also employed. More specifically, a Mixture Model Repeated Measures (MMRM) analysis was employed to model the change in each of the GODDESS scales per anchor group:

- Stable vs Minimally worse
- Stable vs Minimally worse + Much worse
- Stable vs any level of worsening
- Stable vs Minimally improved
- Stable vs Minimally improved + Much improved
- Stable vs any level of improvement

The above models were estimated if there was a sufficient number of patients (i.e., >10 per group).

For this analysis, participants were grouped based on their Cycle 5 anchor result, or in case of missing data at Cycle 5, on their last recorded anchor result. In case a participant was no longer in the trial and the last recorded visit was an off treatment/EOT visit, data from this visit were used. In this case, this visit was awarded a Cycle number equal to the last clinic visit +1 (i.e., if participants last recorded visit was the on-Cycle 3 visit, the off- treatment/EOT visit was coded as Cycle 4). This data treatment preserves the linear trend of time in the MMRM.

The GODDESS PRO scale score changes at Cycles 2 through 5 were used as the dependent variable, with baseline score used as covariate and time (e.g., Cycles 2 through 5) as a categorical variable. Separate models were employed for each of the symptom (i.e., DTSS) and impact (i.e., DTIS) scores, and for each of the stratifications listed above. Both within-group and between-group differences were modeled. The “overall” estimate (rather than the estimate associated with a specific Cycle) was used to inform the meaningful change threshold. Specifically, the “overall” estimates for each group were employed to estimate the within-individual responder definition, and the between-groups estimates to inform the between-groups meaningful change estimate. Of particular importance were the estimates associated with the minimal improvement and minimal worsening groups, and the difference between these groups and the stable group. These estimates were assumed as the minimal amount of change needed to show an improvement. However, sometimes a meaningful change needs to be an estimate that is larger than a minimal change, and therefore estimates from other groups were also considered for triangulation to determine an appropriate meaningful change threshold.

**Results:**

**GODDESS PRO Symptom Scale: DTSS**

Overall estimates from the MMRM analysis for each DTSS change from baseline score are presented below. More specifically, MMRM analysis through Cycle 5 is presented by the Minimally improved and Much improved groups versus Stable group based on PGIS or PGIC anchor (the latter was employed only for DTSS intra-abdominal domain). A within-patient absolute meaningful change threshold value of 1.33 for DTSS total score, 2.07 for DTSS pain domain, and 1.41 for DTSS extra-abdominal domain score using overall value of Minimally and Much improved groups based on PGIS was suggested. All suggested threshold values showed a higher value than Stable group change score, with a difference between 1.16 to 1.51 points. Also, a within-patient absolute meaningful threshold value of 0.65 for Minimally and Much improved groups based on PGIC for DTSS intra-abdominal domain score was suggested, showing a difference of 0.39 scores compared with Stable group (**Appendix Table 7**).

**Appendix Table 7 Overall MMRM estimates per PGIS/PGIC* group - DTSS total and domain scores**

|  | Stable | | | | Minimally/Much Improved | | | | Stable vs Improved |
| --- | --- | --- | --- | --- | --- | --- | --- | --- | --- |
| Score/ Timepoint | **N** | **Mean (SD)** | | **CFB LS Mean (SE)** | **N** | **Mean (SD)** | | **CFB LS Mean (SE)** | **Diff In Mean Change**  **[95% CI]** |
|  |  | **Baseline Value** | **Score Value** |  |  | **Baseline Value** | **Score Value** |  |  |
| Total Score | 55 | 3.31 (2.42) | 2.98 (2.57) | -0.17 (0.18) | 51 | 3.83 (2.45) | 2.45 (2.22) | -1.33 (0.19) | 1.16  [ 0.64, 1.68] |
| Pain Domain | 55 | 3.73 (2.63) | 3.04 (2.75) | -0.56 (0.23) | 51 | 4.16 (2.77) | 2.04 (2.35) | -2.07 (0.23) | 1.51  [ 0.86, 2.16] |
| Extra-abdominal | 55 | 3.26 (2.54) | 2.88 (2.74) | -0.25 (0.21) | 51 | 3.78 (2.61) | 2.31 (2.43) | -1.41 (0.22) | 1.16  [ 0.56, 1.75] |
| Intra-abdominal* | 15 | 1.38 (1.78) | 1.06 (1.42) | -0.26 (0.38) | 14 | 2.62 (2.02) | 1.97 (2.31) | -0.65 (0.40) | 0.39  [-0.78, 1.56] |

For a DTSS scale score (total or domain) to be calculated, it was assumed that at least 4 days out of 7 are available. If sufficient data was not available at baseline, data from the screening period (if available) was used instead. Only subjects with a baseline and at least one post-baseline score were included in the analysis. N is the number of subjects with a baseline and a post-baseline score at each timepoint; CFB: Change from baseline; SD: Standard deviation; LS Mean: Least Square Mean.

*For intra-abdominal score, the PGIC anchor was used.

**GODDESS PRO Impact Scale: DTIS**

Overall estimates from the MMRM analysis for each DTIS domain change score from baseline are presented below. More specifically, MMRM analysis through Cycle 5 is presented by Minimally improved and Much improved groups versus Stable group based on PGIS anchor (**Appendix Table 8**). Within-patient absolute meaningful change threshold values of 0.56 for DTIS physical functioning domain, 0.44 for DTIS sleep domain, and 1.75 for DTIS emotional impact domain based on PGIS Minimally and Much improved groups were suggested. All suggested threshold values showed a higher value than Stable group change score, with a difference of 0.40, 0.31, and 1.18 values, respectively.

**Appendix Table 8 Overall MMRM estimates per PGIS group - DTIS domain scores**

|  | Stable | | | | Minimally/Much Improved | | | | Stable vs Improved |
| --- | --- | --- | --- | --- | --- | --- | --- | --- | --- |
| Score/ Timepoint | N | Mean (SD) | | CFB LS Mean (SE) | N | Mean (SD) | | CFB LS Mean (SE) | Diff In Mean Change  [95% CI] |
|  |  | Baseline Value | Score Value |  |  | Baseline Value | Score Value |  |  |
| Physical Functioning | 58 | 1.69 (1.22) | 1.47 (1.24) | -0.15 (0.07) | 52 | 1.94 (1.11) | 1.29 (1.03) | -0.56 (0.08) | 0.40  [ 0.20, 0.61] |
| Sleep | 58 | 2.04 (1.29) | 1.79 (1.28) | -0.14 (0.10) | 52 | 1.87 (1.33) | 1.41 (1.24) | -0.44 (0.10) | 0.31  [ 0.02, 0.59] |
| Emotional Impact | 58 | 4.23 (2.84) | 3.64 (2.86) | -0.57 (0.20) | 52 | 5.38 (2.60) | 3.43 (2.44) | -1.75 (0.21) | 1.18  [ 0.60, 1.76] |

Only subjects with a baseline and at least one post-baseline score were included in the analysis. N is the number of subjects with a baseline and a post-baseline score at each timepoint; CFB: Change from baseline; SD: Standard deviation; LS Mean: Least Square Mean.

**Appendix 3.**

Countries included in this study are included displayed below followed by a list of the review boards used.

| **Site (anonymized)** | **Patients Randomized** | **Region** |
| --- | --- | --- |
| 1 | 14 | US |
| 2 | 10 | US |
| 3 | 8 | Germany |
| 4 | 7 | Canada |
| 5 | 7 | Belgium |
| 6 | 7 | Netherlands |
| 7 | 7 | US |
| 8 | 6 | US |
| 9 | 5 | US |
| 10 | 5 | US |
| 11 | 5 | US |
| 12 | 5 | US |
| 13 | 5 | US |
| 14 | 5 | US |
| 15 | 5 | Germany |
| 16 | 4 | UK |
| 17 | 4 | US |
| 18 | 4 | US |
| 19 | 3 | US |
| 20 | 2 | US |
| 21 | 2 | UK |
| 22 | 2 | US |
| 23 | 2 | Belgium |
| 24 | 2 | Belgium |
| 25 | 2 | Italy |
| 26 | 2 | Germany |
| 27 | 2 | Italy |
| 28 | 1 | US |
| 29 | 1 | US |
| 30 | 1 | US |
| 31 | 1 | US |
| 32 | 1 | US |
| 33 | 1 | Netherlands |
| 34 | 1 | Italy |
| 35 | 1 | US |
| 36 | 1 | US |
| 37 | 1 | US |

LIST OF IRBS/IECs

| **Name of IRB** | **Address** |
| --- | --- |
| Advarra IRB* | 6940 Columbia Gateway Drive  Suite 110  Columbia, MD 21046 USA |
| Mayo Clinic Institutional Review Board | 200 First Street SW  Suite 201 Building 4-60  Rochester, MN 55905 USA |
| U.T. MD Anderson Cancer Center Institutional Review Board | 7007 Bertner Avenue  Unit 1637  Houston, TX 77030 USA |
| Administrative Panels on Human Subjects in Medical Research ("Stanford IRB"); Research Compliance Office; Stanford University | 1705 El Camino Real  Palo Alto, CA 94306 USA |
| University of Michigan Medical School Institutional Review Board (IRBMED) | 2800 Plymouth Rd  Building 520, Room 3214  Ann Arbor, MI 48109 USA |
| WCG IRB | 1019 39th Avenue SE  Suite 120  Puyallup, WA 98374 USA |
| Cincinnati Children's Hospital Medical Center, Institutional Review Board | 3333 Burnet Avenue  MLC 5020  Cincinnati, OH 45229 USA |
| Duke University Health System IRB Office (DUHS IRB) | Hock Plaza  Suite 405  2424 Erwin Road  Durham, NC 27705 USA |
| Medical College of Wisconsin/ Froedtert Hospital Institution Review Board - Human Research Protection Program | 8701 Watertown Plank Road  HRC-MACC FUND 3040  Milwaukee, WI 53226 USA |
| Oregon Health & Science University Institutional Review Board | 3181 SW Sam Jackson Park Road  L106-RI  Portland, OR 97239 USA |
| University of Miami Institutional Review Board | Human Subject Research Office (M809)  1400 NW 10th Avenue  Suite 1200A  Miami, FL 33136 USA |
| Biomedical Research Alliance of New York, LLC / Institutional Review Board | 1981 Marcus Avenue  Suite 210  Lake Success, NY 11042 USA |
| Memorial Sloan Kettering Cancer Center Institutional Review Board/Privacy Board | 1275 York Avenue  New York, NY 10065 USA |
| Johns Hopkins Medicine Institutional Review Boards | 1620 McElderry Street  Reed Hall  Suite B-130  Baltimore, MD 21205 USA |
| Dana-Farber Cancer Institute Institutional Review Board | 450 Brookline Avenue  OS229  Boston, MA 02215 USA |
| Northwestern University, Office for the Protection of Research Subjects, Institutional Review Board | 750 N. Lake Shore Drive  7th Floor  Chicago, IL 60611 USA |
| University of Pennsylvania Office of Regulatory Affairs | 3600 Civic Center Boulevard  9th Floor  Philadelphia, PA 19104 USA |
| University of Southern California (USC) Institutional Review Board | 1640 Marengo Street  Suite 700  Los Angeles, CA 90033 USA |
| Vanderbilt University Institutional Review Board | 3319 West End Avenue  Suite 600  Nashville, TN 37203 USA |
| UT Southwestern Medical Center Institutional Review Board | 5323 Harry Hines Boulevard  BL9.100  Dallas, TX 75390-8843 USA |
| McGill University Health Centre Research Ethics Board (MUHC) | 2155 Guy Street 2nd Floor  Room 231  Montreal, Quebec H3H 2R9 Canada |
| CHU UCL Namur - Godinne | Avenue Dr. G. Therasse 1  Mont-Godinne, Belgium 5530 |
| Ethikkommission II der Universität Heidelberg (Medizinische Fakultät Mannheim) | Theodor- Kutzer-Ufer 1-3  Haus 42, Ebene 3  68167 Mannheim Germany |
| Comitato Etico IRCCS di Candiolo | Strada Provinciale 142  Candiolo, Torino Italy 10060 |
| MREC NKI-AvL | Plesmanlaan 121  Amsterdam 1066 CX  North-Holland  The Netherlands |
| London - Surrey Borders Research Ethics Committee, Research Ethics Committee (REC) London Centre | Ground Floor  Skipton House  80 London Road  London, United Kingdom SE1 6LH |

**Appendix 4.**

Schedule of assessments


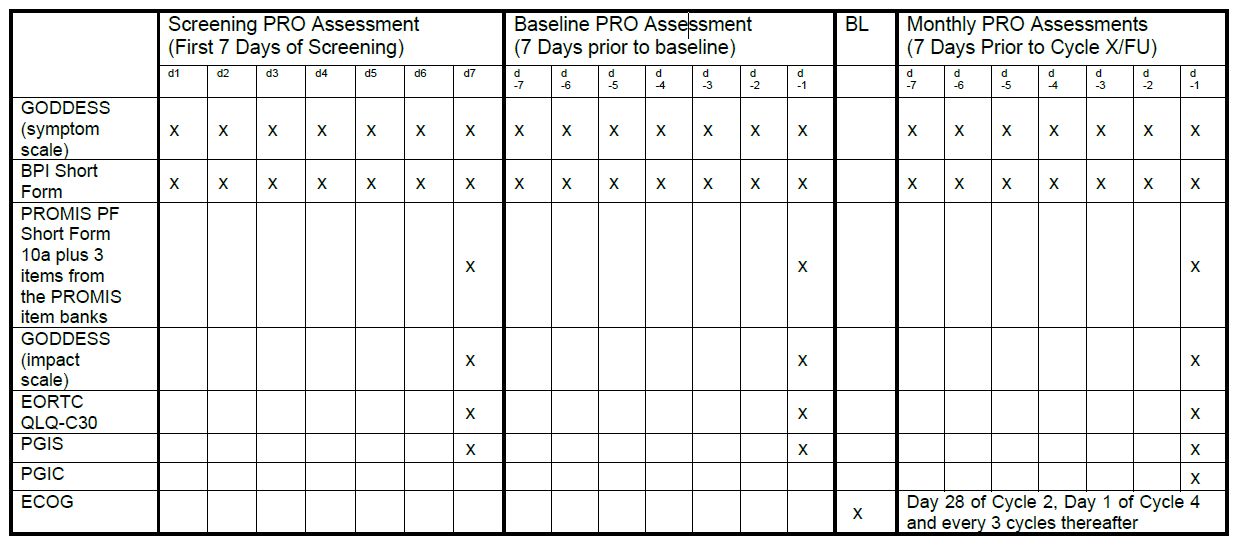

Supplement: Supplemental File1 [file NIHMS1913945-supplement-Supplemental_File1.docx]
